# Hydroxyethylamine & phthalimide analogs restoring defects due to GNE dysfunction: rare disease therapeutic significance

**DOI:** 10.1186/s10020-025-01377-1

**Published:** 2025-12-03

**Authors:** Shagun Singh, Meenakshi Bansal, Neha Sharma, Vikas Yadav, Fluencephila Mashangva, Jyoti Oswalia, Vaishali Gautam, Gagan Deep Jhingan, Naidu Subbarao, Brijesh Rathi, Ranjana Arya

**Affiliations:** 1 School of Biotechnology, Jawaharlal Nehru University, New Delhi, 110067 India; 2 Department of Chemistry, HG Khorana Centre for Chemical Biology, Hansraj College, University of Delhi, Delhi, 110007 India; 3 School of Computational and Integrative Sciences, Jawaharlal Nehru University, New Delhi, 110067 India; 4 VProteomics, Green Park, New Delhi, 110016 India

**Keywords:** GNE myopathy, Rare genetic disease, Therapeutics, Small molecules

## Abstract

Rare diseases refer to a group of neglected diseases with low prevalence that face challenges in diagnostics as well as therapeutics due to phenotypic heterogeneity and ineffective clinical trials. In this study, we evaluated two novel analogs of hydroxyethylamine & phthalimide (LTC-181 and LTC-1717) for their potential effect on the epimerase activity of mutant GNE proteins associated with GNE myopathy. GNE gene encodes a key bifunctional sialic acid biosynthetic enzyme, UDP-N-acetyl Glucosamine 2-epimerase/N-acetyl Mannosamine Kinase; GNE). The compounds have significantly increased the epimerase activity of r-F307C-GNE and r-A555V-GNE mutant proteins in vitro. Reduced GNE epimerase activity and sialic acid content in muscle cell-based model for GNE function (SKM-GNEHz) was increased by 2-fold after addition of these compounds. The proteomic study showed that the compounds affected cytoskeletal organization, autophagy and muscle atrophy. Also, treatment with analogs enhanced the cell viability of SKM-GNEHz cells with increased F-actin polymerization and cell migration, thereby, restoring GNE deficient function. Additionally, effect of these compounds was observed with enhanced autophagy and reduced muscle atrophy function in GNE deficient muscle cell. Docking and interaction studies showed that LTC-1717 stabilize GNE better than LTC-181, indicating better therapeutic potential. Overall, this study indicates that HEA-phthalimide analog could be promising leads for treating GNE myopathy.

**Supplementary Information:**

The online version contains supplementary material available at 10.1186/s10020-025-01377-1.

## Introduction

Rare diseases represent a set of more than 8000 recognized diseases that occur at a prevalence of less than 10/10,000 people. Most of the rare diseases are incurable with adverse effects on the patients and their families. Major challenges in diagnostics and therapeutics of rare diseases include low patient numbers, an incomplete understanding of the pathomechanism, phenotypic heterogeneity, and a lack of effective clinical trials. One of the ultra-rare diseases is GNE Myopathy (GNEM). It is caused by the defect in sialic acid synthesis due to a mutation in the key sialic acid biosynthesis enzyme, GNE (UDP-N-acetylglucosamine2-epimerase/N-acetyl mannosamine kinase). The starting compound for the biosynthesis of sialic acids is UDP-*N*-acetylglucosamine (UDP-GlcNAc). UDP-GlcNAc is converted into N-Acetylmannosamine (ManNAc) with the help of UDP-GlcNAc 2-epimerase. ManNAc is then phosphorylated at C-6 by the N-acetyl mannosamine kinase. This leads to the formation of *N*-acetylneuraminic acid or sialic acid subsequently [19]. The worldwide prevalence of GNEM is 1–9 individuals per million. It is an adult-onset neuromuscular disease characterized by muscle weakness, presence of rimmed vacuoles and protein aggregation.

The participation of patients in clinical trials for rare diseases from low-income countries is very poor [5]. Therefore, developing strategies and identifying new molecules will have a huge impact on people suffering from rare diseases in India and worldwide. Some clinical trials have been executed to evaluate the safety and potency of substrate replacement therapy for treating GNE myopathy. Intravenous Immunoglobulin (IVIg) has been explored as a potential treatment for GNE Myopathy (NCT00195637). But this is rapidly ejected out of the body, and hence, its slow-release formulation – Sialic acid extended release (SA-ER), was tested (NCT01359319). However, the Phase 3 clinical trial conducted by Ultragenyx Pharmaceutical Inc., indicated non-significant effect on muscle strength of GNE Myopathy patients. [32]. On the contrary, Japanese study recently reported significant improvement in GNE Myopathy muscle strength compared to placebo group with SA-ER [50]. Treatment with Sialic acid (SA) precursor, ManNAc, had shown signs of slowing the disease progression in a phase 2 trial (NCT02346461). Now, a multi-centric study exploring the potential of ManNAc in treating GNE Myopathy is under trial. Other small molecules may be explored as potential therapeutic options for GNE Myopathy.

In-depth exploration of GNE’s functions beyond its involvement in sialic acid biosynthesis aims to identify additional therapeutic targets and enhance our understanding of disease pathomechanisms. GNE was shown to interact with α-actinin1/2 and CRMP-1 (Collapsin Response mediator protein), suggesting a direct role of GNE in the cytoskeletal network. Studies on GNE mutant cells revealed the hyposialylation of β1 integrin and its downstream effects showed altered RhoA and cofilin activation, ultimately affecting F-actin polymerization and cell migration [12, 16, 55]. Also, GNE myopathy is characterized by the presence of protein aggregates of β-amyloid, tau, and other proteins. These protein aggregates resulted from protein misfolding in the Endoplasmic Reticulum and improper chaperone function, such as HSP70, calreticulin, PrdxIV etc. [6]. Accumulation of misfolded protein generates ER stress and upregulation of UPR pathway leading to formation of protein aggregates [6, 7]. In general, the protein aggregates are removed in the cell by proteosomal mediated degradation pathway or autophagosomal lysosomal pathway [24]. In GNE myopathy, muscle biopsy samples and in vitro cell based models, upregulation of autophagic markers such as LAMPs (lysosomal associated membrane proteins), p62 and LC3II/I ratio has been reported [38, 41]. However, the mechanism of autophagosome formation in GNE deficient cells is poorly understood. Further the muscle weakness phenotype observed in GNE Myopathy patients has been attributed to upregulation of muscle atrophy markers such as MuRF1 and Atrogin 1 [9]. In addition to this, it has also been reported that GNE plays a role in the regulation of the cell cycle through the DNA damage-repair pathway [20). In vitro GNE myopathy model using patient-derived induced pluripotent stem cells (iPSCs) shows impaired myogenesis [45]. Thus, the development of drug for GNE Myopathy may target cytoskeletal network, ER stress, protein aggregation, autophagy and muscle atrophy as some of the characteristic pathways affected in GNE deficient cells.

Recent Studies explored hydroxyethylamine (HEA) derivatives as a synthon for the discovery of anti-malarial, anti-fungal, HIV protease inhibitors and anti-Alzheimer’s etc. HEA based compounds have been placed in early phase clinical trials for anti-Alzheimer activity [53]. Also, phthalimide derivatives are known to possess numerous biological activities including anti-cancer, anti-inflammatory and anti-epileptic. Both the pharmacophores have been explored for the design and development of new pharmaceuticals for various diseases such as schistosomiasis [48], malaria [3, 7], leishmaniasis [29], tuberculosis [42], and dengue [18]. It is noteworthy that the Food and Drug Administration (FDA) has approved three drugs, namely thalidomide (1998) [10], lenalidomide (2006) [30], and pomalidomide (2013) [52], which possess Pht scaffold for human use. When initially evaluated, several potential drug candidates failed to demonstrate any apparent biological effects [47]. We screened additional compounds of the LTC chemical library to broaden this search. While the other candidates were not significant, LTC-1717 and LTC-181 showed quantifiable effects on epimerase activity. These initial findings led us to choose LTC-1717 and LTC-181 for in-depth analysis in this investigation. We determined the effect of these compounds on in vitro GNE activity as well as alternate cellular roles of GNE such as Cell Migration, F-actin polymerization, ER stress, Protein Aggregation, Autophagy, and Muscle Atrophy. Our study demonstrated that these drugs positively affected the GNE epimerase activity, possibly via direct binding, and contributed significantly to overcoming GNE dysfunction. Recent studies have also shown that some of the small effector molecules, such as CGA regulating cofilin function for actin polymerization, BGP-15 activator for Chaperone HSP70, and IGF-1 ligand for IGF-1R showed improved GNE-related function [37, 40). Thus, the identification of novel HEA-Pht analogs present a promising therapeutic lead for the design of drugs for GNE Myopathy, a rare neuromuscular genetic disorder.

## Materials and methods

### Sources of materials and chemicals

Animal tissue culture components and Fetal Bovine Serum (FBS) were sourced from HiMedia, India. All plasticware was acquired from Corning, USA. Transfection reagents Opti-MEM and Lipofectamine 3000 were procured from Invitrogen, USA. Hoechst nuclear stain and TRITC-Phalloidin (Cat. no. P1951) were purchased from Sigma-Aldrich, USA. PVDF membranes were obtained from MDI, India. GlcNAc and ManNAc were purchased from Sigma-Aldrich. Other chemicals were sourced from SRL and Merck in India, Sigma-Aldrich, and Fisher Scientific, USA. The pENTR1A Plasmid containing MERO-GFP was generously provided by Prof Fumihiko Urano from the Department of Pathology and Immunology at Washington University School of Medicine in St. Louis, MO, USA.

### Compound synthesis and spectroscopic data

#### (S)-N-((2 S,3 S)−4-(4-benzylpiperazin-1-yl)−3-hydroxy-1-phenylbutan-2-yl)−2-phenylpropanamide (LTC-181)

Briefly, epoxide, (2R,3 S)−3-(t-BOC)amino-1,2-epoxy-4-phenylbutane (1) (0.019 mol), benzylpiperazine (2) (0.019 mol), and 5 mL of ethanol placed in a 50 mL round-bottom flask was heated using microwave irradiations. The reaction mixture was cooled to RT before the solvent was concentrated at a low pressure. TLC was used to check for reaction completion, and the contents were then concentrated under vacuum to remove the solvent, resulting in a BOC-protected compound (3), which was then dissolved in dichloromethane (DCM) and, after the addition of 20% trifluoroacetic acid (TFA), the contents were stirred for 1 h to secure the deprotected compound (4). To eliminate excess TFA, the organic layer was washed with DCM. The resulting mixture was rinsed with 5% NaHCO3 to neutralize the acid. In the next step, the carboxylic acid (5) (1.0 mmol) dissolved in DCM was taken in an RB flask, and triethylamine (TEA; 0.0146 mol) was added dropwise. After 20 min, EDC·HCl (0.0132 mol) was added, followed by the addition of HOBt (0.0132 mol) after another 20 min. Following 30 min of stirring at 0 °C, the deprotected intermediate (4) (1.0 mmol) was separately dissolved in dichloromethane and added to the reaction flask, followed by stirring at RT for 24 h. The solvent was removed, and the contents were extracted with ethyl acetate. The organic layer was washed with water, then brine solution, and dried over sodium sulphate. The obtained product was purified by Flash column chromatography in a methanol/chloroform (1:49, v/v) solvent solution. LTC-181 exhibited a maximum absorbance (λmax) in methanol at 210 nm in the UV-Vis spectrum.

Spectroscopic Data: White solid; yield, 82%; m.p. 105–106 °C; ^1^H NMR (400 MHz, CDCl_3_) δ 7.29–7.21 (m, 6 H), 7.20 (d, J = 5.0 Hz, 5 H), 7.15 (s, 3 H), 7.11–7.04 (m, 1H), 5.79 (d, J = 30.0 Hz, 1H), 3.95 (t, J = 12.6 Hz, 1H), 3.58–3.48 (m, 1H), 3.45–3.33 (m, 3 H), 2.88–2.68 (m, 2 H), 2.51 (d, J = 24.7 Hz, 1H), 2.36 (d, J = 34.6 Hz, 5 H), 2.18 (d, J = 7.1 Hz, 1H), 2.12–1.98 (m, 2 H), 1.94–1.81 (m, 1H), 1.37 (s, 3 H). ^13^C NMR (100 MHz, CDCl_3_) δ 173.9, 141.8, 138.2, 137.8, 129.8, 129.4, 128.9, 128.4, 127.6, 127.2, 126.4, 77.6, 77.5, 77.0, 65.3, 62.9, 60.1, 52.8, 51.4, 47.2, 38.9, 18.2. (HRMS) (ESI-TOF) m/z: [M + H]^+^ Calcd. for C30H37N3O2, 472.2886; found, 472.2984.

#### 2-(2-Hydroxy-3-(4-(2-hydroxyethyl)piperazin-1-yl)propyl)isoindoline-1,3-dione (LTC-1717)

Following the previously reported procedure by our group [26] 2-(oxiran-2-ylmethyl)isoindoline-1,3-dione (6) (1.0 mmol), secondary amines (7) (1.0 mmol), ethanol (1 mL) were taken in a pressure seal tube and heated under microwave at 200 °C for 0.5-1.0 min. The workup of the obtained crude product after removing the excess ethanol was carried out in ethyl acetate (20 mL x 3) and brine solution (15 mL x 3). The organic layer was dried over anhydrous sodium sulphate. The excess ethyl acetate was removed on a rotary evaporator, and the product was purified either by washing with hexane or recrystallizing with ethyl acetate and hexane in 1:9 ratios. ^1^H NMR, ^13^C NMR, and HR-MS techniques confirmed the chemical composition of LTC-1717.

Spectroscopic Data: White solid; Yield: 88%; m.p. 107–109 ℃; ^1^H-NMR (400 MHz, DMSO-d6) δ 7.84–7.78 (m, 4 H, H-1 and 2) 4.93 (s, 1H, OH), 4.28 (s, 1H, OH), 3.91 (s, 1H, H-3), 3.63–3.49 (m, 2 H, H-4), 3.34 (t, J = 6.4 Hz, 2 H, H-5), 2.43–1.92 (m, 12 H, H-6 to 9). ^13^C-NMR (100 MHz, DMSO-d6) δ 168.6 (C = O), 134.7 (C-1), 132.4 (C-1a), 123.4 (C-2), 64.0 (C-3), 63.6 (H-8), 60.7 (H-5 and 9), 58.8 (H-7), 53.4 (H-6), 43.6 (H-4). HRMS calcd. (M + H) for C17H23N3O4 333.1689; found 333.1672. HPLC purity: 98.3%.

### Bacterial strain, culture conditions and purification of GNE protein

Recombinant wild type GNE (r-wtGNE) (hGNE1; NM_005476) and mutations (r-F307C-GNE, and r-A555V-GNE) were generated by site-directed mutagenesis in pcDNA3.1 vector followed by sequence confirmation [7]. The cells were cultured and the protein was purified as described by Sharma et al. [47].

### Cell lines and preparation of whole-cell lysate

Our laboratory established an L6 skeletal muscle cell-based model system termed SKM-GNEHz, characterized by heterozygous knockout of GNE exon 3 via homologous recombination (IPO No 511763). These cell lines exhibited reduced GNE epimerase activity and sialic acid content. The cells were cultured in T-25 flasks containing Dulbecco’s Modified Eagle Medium (DMEM) at standard conditions. Then the cells were lysed using RIPA lysis buffer (20 mM Tris–HCl (pH 7.5), 1% NP-40, 150 mM NaCl, 1 mM EDTA, and a protease inhibitor cocktail). After 45 min incubation on ice, the cell lysates were centrifuged at 13,000 rpm for 10 min to remove cellular debris.

### Antibodies

Primary antibodies for anti-LC3A/B (CST, Cat no. 4108 S) and anti-p62 (CST, Cat no. 5114 S), were purchased from Cell Signaling Technology, USA. Anti-Murf1 (sc-398608), anti-GAPDH (sc-32233), and HRP conjugated secondary antibodies for immunoblotting were purchased from Santa Cruz Biotechnology, USA. Alexa Fluor tagged secondary antibodies were purchased from Invitrogen, USA.

### Epimerase enzyme activity

Assay was performed by Morgan and Elson method, as described [44, 47]. Equal concentrations of protein were incubated with 45 mM Na_2_HPO_4_ (pH 7.5), 10 mM MgCl_2_, and 1 mM UDP-GlcNAc to a final volume of 40 µl. The enzymatic reaction was conducted at 37 °C for 30 min and terminated by boiling for 1 min. ManNAc was quantified using the standard graph [44].

### Sialic acid content

Sialic acid levels were assessed using an adapted periodate/resorcinol method [37]. Cells were cultured in serum free media for 24 h and harvested. The cells were lysed via three freeze/thaw cycles in lysis buffer. Total protein content was determined using the Bradford assay. Sialic acid content was determined as described before [14].

### TRITC -phalloidin staining

In 6-well culture plates, 0.5 × 10^5^ cells were cultured on sterile coverslips in serum free media for 24 h. After growing cells were treated as per the protocol described by Devi et al., [14].

### Cell migration assay

0.5 × 10^5^ cells were seeded in 6 well plate and incubated at 37 °C for 24 h in serum free media. Subsequently, the cells were treated as described by Mashangva et al., [37].

### Cell proliferation

Cells were plated in 96-well tissue culture plates at a seeding density of 3000 cells per well in serum free media and cultivated for up to 48 h. MTT (SRL, India) was added to at a concentration of 0.5 mg/mL. Following a 3 h incubation period. After removing MTT, 100 µl of DMSO was added and incubated for 10 min. The absorbance was then taken at 570 nm using SpectraMaxplus 384 (Molecular Devices, USA).

### Crystal violet staining test

Cells were seeded in 96-well tissue culture plates at a seeding density of 8000 cells/well in serum free media. After the incubation time (24–48–72 h), the medium was removed, the cells were fixed with 10% buffered formalin for 20 min at RT and then stained using a 1% v/v solution of Crystal Violet for 30 min. Acetic acid was added to elute the dye, and absorbance was read at 560 nm using SpectraMax plus 384 (Molecular devices, USA).

### Immunoblotting

Whole cell lysates resolved on SDS-PAGE were transferred to PVDF membranes. The membranes were blocked with 5% skimmed milk powder in 1X TBST for 2 h and probed with primary antibody overnight at 4 °C. The blots were incubated with HRP-conjugated secondary antibody for 45 min at RT and developed using enhanced chemiluminescent HRP substrate (Millipore, USA/BioRad) in ChemiDoc (BioRad).

### Confocal microscopy

10^4^ cells were seeded on the coverslip in DMEM with 10% FBS, after 24 h DMEM was replaced with DMEM without serum and grown further for 24 h. Then media was removed and cells were washed with 1× PBS (pH 7.4) twice followed by fixation with 3.7% paraformaldehyde for 20 min. Permeabilization and blocking were done using 0.1% TritonX-100 in 1% BSA in 1X PBS (pH 7.4) for 10 min. Followed by washing twice with 1× PBS (pH 7.4). Then overnight incubation with the primary antibody in a moist chamber at 4°C. Cells were then incubated with the fluorophore‐tagged secondary antibody for 45 min followed by washing with 1× PBS (pH 7.4) three times. Mounting was done by using 10 mg/mL DABCO prepared in 90% glycerol. Confocal images were visualized under Olympus Fluoview FV1000 laser scanning microscope.

### Treatment with small molecules

After growing cells in serum free media for 24 h, the cells were treated with 1 µM LTC-181 or 1 µM LTC-1717 for another 24 h before harvesting the cells.

### In-silico analysis

The *Homo sapiens* GNE structures were retrieved from RCSB (PDB IDs: 4ZHT, 3EO3). Mutations (Phe307Cys in the epimerase domain, Ala555Val in the kinase domain) were introduced using UCSF Chimera. Energy minimization (Schrödinger) and 300 ns MD simulations (GROMACS) were performed, and the lowest-energy protein conformations were extracted.

#### Protein preparation, binding site and grid generation

The proteins were prepared using protein preparation wizard of Schrödinger suit (version 2019.1) [34] The active site residues for 3EO3(ASN516, ASP517, GLU566, HIS569, GLU588), and for 4ZHT (ARG19, SER23, ARG113, ARG321, HIS220, ASN253, VAL282, SER301, SER302, GLU307) were identified through PyMOL, LigPlot analysis, and literature (UDP-GlcNAc substrate-binding pocket of the GNE epimerase domain). Ligands (LTC-1717, LTC-181, UDP-GlcNAc; PubChem ID 9547196) were prepared using the LigPrep module in Schrödinger.

#### Molecular docking based virtual screening

The GLIDE module of Schrödinger suite version (2019.1) was used to perform molecular docking [15, 17]. Top-ranking molecules from GLIDE XP outputs were further evaluated using GOLD Suite (version 5.2) [21] and binding energies predicted with the X-score consensus scoring function [54]. Protein-ligand complexes were visualized by Pymol and Ligplot [28].

#### Molecular dynamics simulations

We performed molecular dynamics (MD) simulations using GROMACS (version 5.1.2) [1]. Simulations ran for 300 nanoseconds (15,000,000 steps) with the protein parameterized using the AMBER99SB force field and ligand topology generated via GAFF and BCC charges using the ACPYPE server [23, 56]. Binding free energy of the complexes was calculated using the MM-PBSA (Molecular Mechanics/Poisson-Boltzmann Surface Area) method. Remaining procedures followed previously established protocols [25, 27].

### Surface plasmon resonance

Surface Plasmon resonance spectroscopy was performed to monitor the interaction of small molecules LTC-1717 and LTC-181 with GNE protein. Immobilization of GNE was done on the activated gold sensor chip. Different concentrations of LTC-1717 and LTC-181 were injected over the chip surface in order to monitor the interaction both for the association and dissociation throughout 500 s using the Auto LAB ESPRIT SPR instrument (Kinetic Evaluation Instruments BV, The Netherlands) with an open cuvette system along with electrochemical resonance facility. PBS was used for immobilization and binding solution. The surface was regenerated with 50 mM NaOH. Data was analyzed using Auto Lab ESPRIT Kinetic evaluation software.

### Proteomics

Protein per sample was used for digestion and reduced with 5 mM TCEP and further alkylated with 50 mM iodoacetamide and then digested with Trypsin (1:50, Trypsin/lysate ratio) for 16 h at 37 °C. Digests were cleaned using a C18 silica cartridge to remove the salt and dried using a speed vac. The dried pellet was resuspended in buffer A (2% acetonitrile, 0.1% formic acid). For mass spectrometric analysis, all the experiments were performed on an Easy-nLC-1000 system (Thermo Fisher Scientific) coupled with an Orbitrap Exploris 240 mass spectrometer (Thermo Fisher Scientific) and equipped with a nanoelectrospray ion source. 1ug of PhosphoPeptides sample was dissolved in buffer A containing 2% acetonitrile 0.1% formic acid and resolved using Picofrit column (1.8micron resin, 15 cm length). Gradient elution was performed with a 0–38% gradient of buffer B (80% acetonitrile, 0.1% formic acid) at a flow rate of 500nl/min for 96 min, followed by 90% of buffer B for 11 min and finally column equilibration for 3 min. Orbitrap Exploris 240 was used to acquire MS spectra under the following conditions: Max IT = 60ms, AGC target = 300%; RF Lens = 70%; *R* = 60 K, mass range = 375 − 1500. MS2 data was collected using the following conditions: Max IT = 60ms, *R* = 15 K, AGC target 100%. MS/MS data was acquired using a data-dependent top20 method dynamically Choosing the most abundant precursor ions from the survey scan, wherein dynamic exclusion was employed for 30s. All samples were processed and RAW files generated were analysed with Proteome Discoverer (v2.5) against the Uniprot Rattus norvegicus database. For dual Sequest and Amanda search, the precursor and fragment mass tolerances were set at 10 ppm and 0.02 Da, respectively. The protease used to generate peptides, i.e. enzyme specificity was set for trypsin/P (cleavage at the C terminus of “K/R: unless followed by “P”). Carbamidomethyl on cysteine as fixed modification and oxidation of methionine and N-terminal acetylation were considered as variable modifications for database search. Both peptide spectrum match and protein false discovery rate were set to 0.01 FDR. The protein identification matrix generated in Proteome Discoverer was exported for downstream statistical analysis, and abundance values were standardized by log2 transformation. Proteins with more than 30% missing values per experimental condition were excluded, while remaining missing intensities were imputed. Data were further normalized by median centering to minimize technical variation while preserving biological differences. The effectiveness of normalization was evaluated using boxplots. Differential protein abundance between groups was determined using the LIMMA framework, with significance defined at p < 0.05. Proteins identified as significant were subsequently analysed for functional enrichment using Gene Ontology (GO) and Reactome pathway analysis (clusterProfiler, ReactomePA) to identify enriched biological processes and signalling pathways. Results were visualized through heatmaps, volcano plots, and enrichment plots for GO terms and pathways. All statistical and visualization analyses were conducted in R (v4.3.2). Gene Ontology (GO) and pathway enrichment analyses were performed to interpret the biological relevance of differentially abundant proteins. Proteins that passed the significance threshold (p-value < 0.05) were subjected to enrichment using the clusterProfiler and ReactomePA R packages. Over-representation analysis (ORA) was conducted across the three GO domains—Biological Process (BP), Molecular Function (MF), and Cellular Component (CC)—as well as Reactome pathway databases. Background sets consisted of all quantified proteins in the study to control for dataset-specific biases. Enrichment significance was determined using the hypergeometric test. Results were reported with p-values and mapped protein counts for top-10 significant pathways/GO terms. Visualization of enriched GO-categories and pathways was carried out using bar plots and dot plots, respectively.

### Statistical analysis

All experimental results were presented as mean ± standard deviation (S.D.) and were derived from a minimum of three independent experiments. GraphPad Prism 9 software was utilized for the statistical analysis. Statistical significance among experimental groups was determined using a Kruskal-Wallis test followed by Dunn’s multiple comparison post-test, with a significance threshold set at *p* < 0.05. Significance levels were denoted as follows: *p < 0.05, **p < 0.01, ***p < 0.001, and ****p < 0.0001 while “ns” indicated non-significant differences.

## Results

### Compound synthesis

**Fig. 1:**
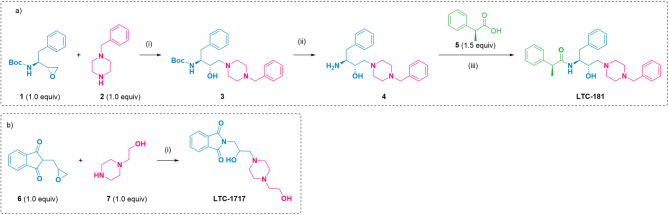
Chemical synthesis. **A** Synthesis of LTC-181; reaction conditions: (i) ethanol, MW, 80 °C, 30 min; (ii) 20 mol% TFA, DCM, 0 °C, 1 h; (iii) EDC·HCl (1.5 equiv), HOBt (1.5 equiv), TEA (2.0 equiv), DCM, 0 °C–rt.; **B** Synthesis of LTC-1717; reaction conditions: i) ethanol, MW, 80 °C, 30 min

HEA and Pht are critical scaffolds in drug discovery due to their unique structural and functional properties. HEA derivatives are valued for their potential to improve pharmacological features such as potency, selectivity, and metabolic stability, making them suitable starting points for designing new therapeutic molecules [22]. As indicated in the literature studies, HEA functionalized with piperazines is an essential scaffold for discovering therapeutic molecules [49]. Pht-based compounds exhibit diverse bioactivities and are attractive synthons for drug discovery and development. Pht features a bicyclic aromatic heterocycle system. Its lipophilic and neutral properties enable its derivatives to efficiently traverse biological membranes [57]. The fusion of Pht and HEA moiety is not explored without a linker, and a series of compounds were synthesized, however, synthetic scheme of two novel compounds, LTC-181 and LTC-1717, possessing HEA-piperazine and HEA-Pht scaffolds, respectively, is illustrated in Fig. 1. In addition to having a piperazine core, a unique scaffold that is known to improve solubility and protein binding is attached, both LTC-1717 and LTC-181 also have hydroxyl substituents that facilitate hydrogen bonding and resemble sialic acid intermediates. Their pharmacophores, however, are very different. LTC-1717 has a phthalimide (Pht) moiety attached to a hydroxyethyl-substituted piperazine. The phthalimide core, flexible linker, and OH group provide stiffness. The HEA (hydroxyethylamine) backbone of LTC-181, on the other hand, is a peptidomimetic pharmacophore with stereocenters that contains a bulky phenylpropanamide group and a benzyl-substituted piperazine. Therefore, LTC-181 offers stereospecificity and hydrophobic contacts, enabling complementary forms of interaction with the target enzyme, while LTC-1717 stresses flexibility and polar interactions. The chemical composition of synthesized compounds was confirmed by the spectroscopic and spectrometry techniques (Figure S1-S7; Supporting Information). All the compounds were tested for functional enhancement of GNE activity by epimerase assay, however, except LTC-181 and LTC-1717, no other compound showed significant enhancement of GNE activity (Figure S8; Supporting Information). Therefore, the study was performed with LTC-181 and LTC-1717 for further analysis. The dose dependent study from a range of 0.25 µM to 20 µM of LTC-1717 and LTC-181 indicated maximum epimerase activity of GNE at 1 µM concentration (Figure S9; Supporting Information).

####  Binding of HEA-Pht analogs with GNE

To investigate the role of LTC-1717 and LTC-181 in regulating GNE function, their direct binding to GNE was assessed using computational docking, MD simulations, and SPR analysis.

#### Molecular docking

**Fig. 2:**
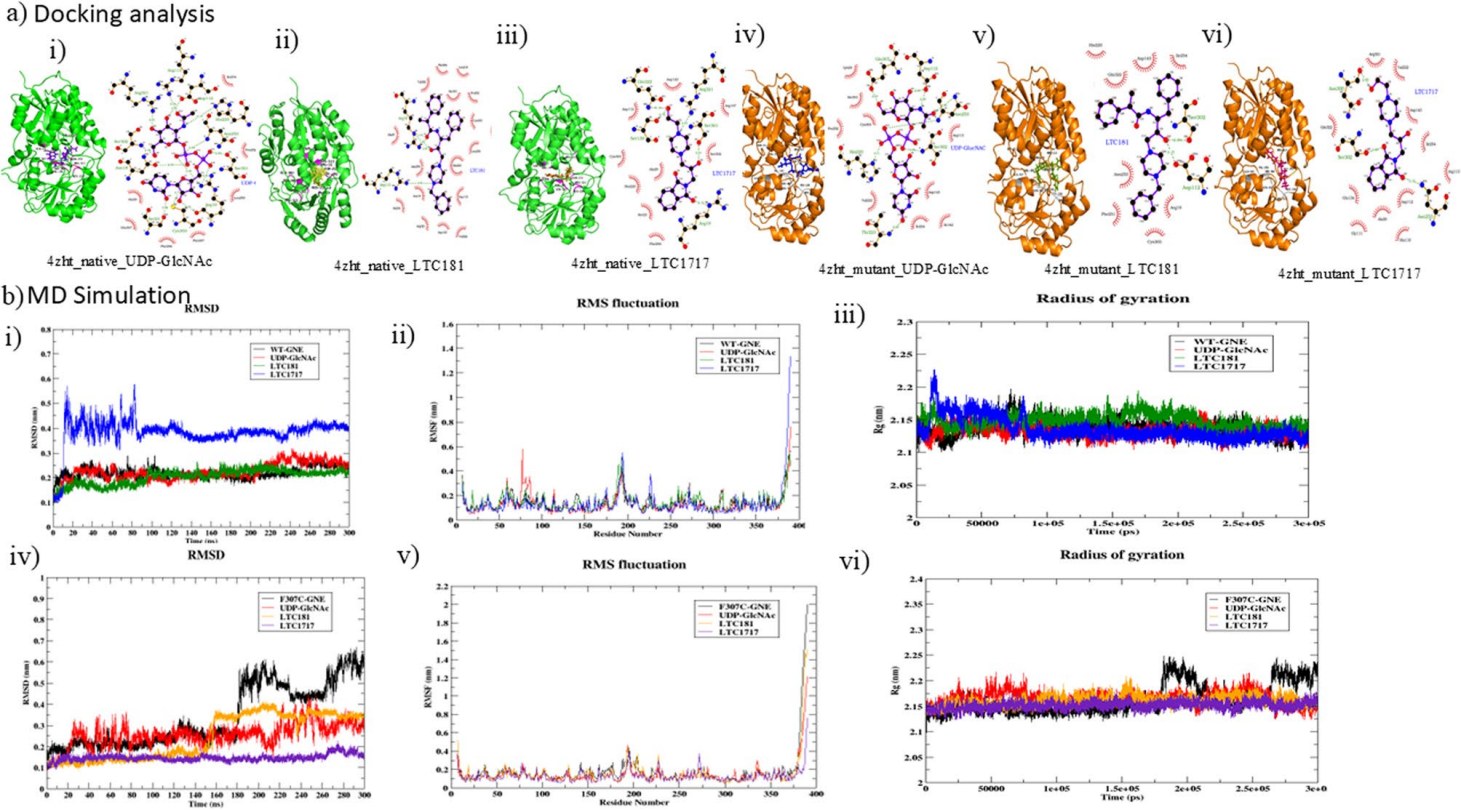
Insilico analysis of small molecule interaction with GNE: **a** Ligplot analysis and docking results of WT GNE protein and mutant F307C-GNE protein with various compounds. **b** RMSD, RMSF and radius of gyration. (i) GlcNAc(9547196) known substrate with WT GNE protein (ii) LTC-181 with WT GNE protein (iii) LTC-1717 with WT GNE protein. (iv) GlcNAc(9547196) with Mutant (F307C-GNE) protein (v) LTC-181 with mutant (F307C-GNE) protein (vi) LTC-1717 with mutant (F307C-GNE) protein

The docking results for the Epimerase domain (4ZHT) in both wtGNE and mutant (F307C-GNE) forms showed differences in ligand binding affinities (Fig. 2a). With the wtGNE, UDP-GlcNAc had the strongest binding with a Glide Score of −14.920 Kcal/mol, while LTC-1717 and LTC-181 exhibited moderate binding (Table 1). With the mutant form, UDP-GlcNAc and LTC-1717 showed reduced affinities, but LTC-181 maintained strong binding potential with a Gold Fitness Score of 79.67 (Table 2). Binding affinity predictions using Xscore supported these findings. UDP-GlcNAc had the most favorable Xscores across both forms (−8.88 kcal/mol and − 6.99 kcal/mol for wtGNE; −8.25 kcal/mol and − 7.50 kcal/mol for F307C-GNE). LTC-1717 showed moderate affinity with wtGNE (−7.82 kcal/mol and − 6.68 kcal/mol) and improved binding with F307C-GNE (−7.11 kcal/mol and − 7.42 kcal/mol). LTC-181 demonstrated strong binding to wtGNE (−10.26 kcal/mol and − 9.50 kcal/mol) and moderate binding with F307C-GNE (−8.77 kcal/mol and − 9.24 kcal/mol). Overall, the mutation generally reduced ligand binding affinity, but LTC-181 exhibited more favorable interactions with the mutant protein, highlighting its potential as a strong binding ligand. In our study, the docking was carried out on the epimerase (4ZHT) separately, since no experimentally resolved structure of full-length GNE is currently available. To mitigate the Limitations of using individual domains, we performed 300 ns MD simulations of both wild-type and mutant (F307C-GNE; A555V-GNE), followed by MM-PBSA free energy calculations. This allowed us to evaluate ligand binding stability in functional region of GNE.

**Table 1:** Docking and post docking analysis of epimerase domain wild type (4ZHT) comparing with benchmark UDP-GlcNAc

S.NO	ID	GlideScore (Kcal/mol)	Xscore (Glide) (Kcal/mol)	Gold Fittness Score	Xscore (Gold) (Kcal/mol)	Hydrogen Bonds	Hydrophobic Interactions	Non-Bonded
1	UDP-GlcNAC	−14.920	−8.88	88.30	−6.99	13	7	56
2	LTC-1717	−6.723	−7.82	52.06	−6.68	5	9	47
3	LTC-181	−6.088	−10.26	75.95	−9.50	3	17	77

**Table 2:** Docking and post docking analysis of epimerase domain mutant (4ZHT) comparing with benchmark UDP-GlcNAc

S.NO	ID	GlideScore (Kcal/mol)	Xscore(Glide) (Kcal/mol)	Gold Fittness Score	Xscore(Gold) (Kcal/mol)	Hydrogen Bonds	Hydrophobic Interactions	Non-Bonded
1	UDP-GlcNAC	−11.880	−8.25	82.98	−7.50	8	9	51
2	LTC-1717	−5.776	−7.11	55.21	−7.42	3	11	41
3	LTC-181	−4.957	−8.77	79.67	−9.24	2	11	50

#### MD simulation and binding free energy calculation

A 300 ns molecular dynamics (MD) simulation using GROMACS 2019 was performed for all protein-ligand complexes, including wtGNE and mutant F307C-GNE, to assess macromolecule dynamics and stability (Figure S12; Supporting Information). Root Mean Square Deviation (RMSD) analysis revealed that wtGNE and its complexes with UDP-GlcNAc, LTC-181, and LTC-1717 had average RMSD values of 0.219 nm, 0.224 nm, 0.203 nm, and 0.383 nm, respectively (Fig. 2b) (i)). LTC-1717 showed higher deviation (peaking at 3.5 Å from 10 to 80 ns) before stabilizing, while LTC-181 demonstrated greater stability, outperforming UDP-GlcNAc. For mutant F307C-GNE, average RMSD values for unbound protein, UDP-GlcNAc, LTC-181, and LTC-1717 were 0.338 nm, 0.254 nm, 0.244 nm, and 0.146 nm, respectively (Fig. 2b) (iv)). LTC-1717 maintained the lowest and most stable RMSD, suggesting superior binding and stability over UDP-GlcNAc and LTC-181, even in the mutated form.

Root Mean Square Fluctuation (RMSF) analysis indicated significant dynamics at the N-terminal, C-terminal, and loop regions, while binding site residues remained stable, highlighting their role in ligand interaction and catalytic activity (Fig. 2b) (ii) & (iv)).

Radius of gyration (Rg) values further supported these findings, showing compactness and rigidity of protein-ligand complexes. For wtGNE, average Rg values for unbound protein, UDP-GlcNAc, LTC-181, and LTC-1717 were 2.1372 nm, 2.131 nm, 2.148 nm, and 2.135 nm, respectively (Fig. 2b) (iii)). For F307C-GNE, Rg values ranged from 2.151 nm to 2.165 nm, with LTC-1717 (Fig. 2b) (vi)) exhibiting the lowest and most stable Rg, underscoring its ability to stabilize the mutant protein and resist unfolding.

Binding Free Energy **(**MM-PBSA) calculations quantified the stability of these complexes by considering van der Waals, electrostatic, and solvation energies. For wtGNE, UDP-GlcNAc showed a binding energy of −65.722 ± 1.764 kJ/mol, while LTC-181 displayed the most favorable energy at −103.547 ± 1.219 kJ/mol, driven largely by van der Waals interactions. In the mutant F307C-GNE, UDP-GlcNAc’s binding energy decreased to −39.891 ± 2.169 kJ/mol, whereas LTC-1717 exhibited a stronger binding energy of −86.668 ± 1.058 kJ/mol. These results highlight LTC-181 and LTC-1717 as stabilizing ligands for both wtGNE and its mutant form, with interactions dominated by electrostatic and van der Waals forces (Table 3). These dynamic properties may be responsible for stronger functional rescue in enzymatic and cellular assays.

**Table 3:** Free energy calculation using mmpbsa of epimerase domain (native and mutant)

	S.No	Complexes	ΔG bind (kJ/mol) (with standard deviation)	ΔG ele (kJ/mol)	ΔGvdw (kJ/mol)	ΔG polsov (kJ/mol)	ΔG SASA (kJ/mol)
Native	1	UDP-GlcNAC	−65.722 +/−1.764	−624.496 +/−2.411	−214.992 +/−1.027	798.117 +/−2.052	−24.476 +/−0.047
	2	LTC-181	−103.547 +/−1.219	−39.212 +/−0.707	−239.585 +/−0.750	202.592 +/−1.435	−27.423 +/−0.075
	3	LTC-1717	−29.062 +/−0.889	−72.989 +/−0.716	−170.528 +/−0.631	235.290 +/−1.075	−20.791 +/−0.045
Mutant	4	UDP-GlcNAC	−39.891 +/−2.169	−550.347 +/−3.407	−209.167 +/−1.172	743.990 +/−3.471	−24.510 +/−0.049
	5	LTC-181	−63.832 +/−1.317	−53.059 +/−0.859	−202.160 +/−0.818	216.568 +/−1.877	−25.153 +/−0.075
	6	LTC-1717	−86.668 +/−1.058	−60.651 +/−0.906	−164.325+/−0.724	155.270 +/−1.566	−17.036 +/−0.054

Whether binding of LTC-1717 and LTC-181 to GNE affected the allosteric site function was further investigated by docking analysis comparison to allosteric inhibitor, CMP-Sialic acid (CMP-SA). Our study showed that LTC-1717 bind weakly in a different orientation to GNE compared to CMP-SA (Figure S13, Supporting Information). This may enhance the biological activity even though the binding affinity is very low of designed ligands suggesting binding affinity alone does not always predict functional efficacy rather conformational stabilization of the mutant protein can be the key determinant of rescue activity. Thus, increased stability guided us to further explore the potential of these molecules in improving cellular functions.

#### SPR analysis

**Fig. 3:**
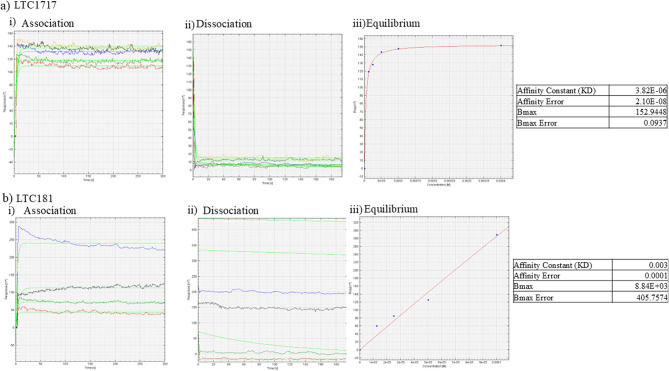
Representative surface plasmon resonance (SPR) data. Dissociation constants (KD), and Bmax were determined by SPR. **a** Graph showing surface plasmon resonance data where increasing concentrations of LTC-1717 (in µM) was injected over the surface containing immobilized GNE protein, **b** Graph showing surface plasmon resonance data where increasing concentrations of LTC-181 (in µM) was injected over the surface containing immobilized GNE protein. Analysis of spectra was done using autolab ESPRIT kinetic evaluation software

LTC-1717 demonstrated a KD value of 3.82E-06 M, indicating moderate affinity binding to the wtGNE Protein. In contrast, LTC-181 exhibited a KD value of 0.003 M, reflecting low affinity binding. These findings suggest that LTC-1717 forms a tight and stable complex with the target protein, while LTC-181 interacts with lower affinity. The differential binding affinities observed between LTC-1717 and LTC-181 may have important implications for their therapeutic potential. LTC-1717 with its higher affinity for the target protein, may offer a greater efficacy and potency in modulating protein function compared to LTC-181 (Fig. 3).

#### Effect of small effector molecules on GNE epimerase activity and Sialic acid content

**Fig. 4:**
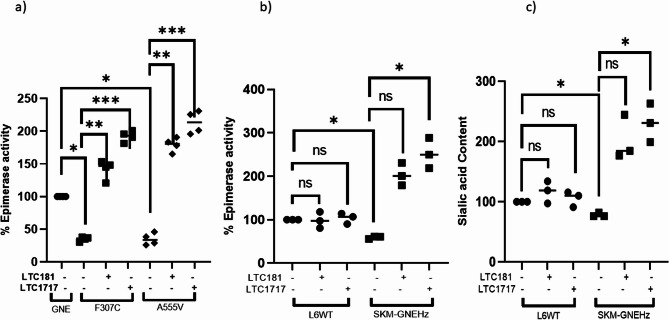
Determination of Epimerase Activity and Sialic acid content: **a**) In vitro Epimerase Activity – Recombinant wild type GNE and mutant protein purified from E. coli was used to determine the epimerase activity in presence of small molecules (LTC-181 & LTC-1717) using Morgan Elson method. N-acetyl-mannosamine was used as a standard. n = 4 Biological Replicates **b**) GNE Epimerase Activity from L6 WT and SKM-GNEHz (knock out cells) in presence of small molecules (LTC-181 & LTC-1717). **c**) Total sialic acid content-The cell lysates of L6 WT and SKM-GNEHz were subjected to periodic acid and resorcinol treatment as described in Methods. The statistical significance was assessed by Kruskal-Wallis test followed by Dunn’s post-test and bars represent the mean ± SD; *n* = 3 Biological Replicates/Triplicates. *p < 0.05, **p < 0.01, ***p < 0.001 and ****p < 0.0001. ‘ns’ represent non-significant

To assess the effect of HEA-Pht analogs on GNE epimerase activity, recombinant GNE protein was expressed and isolated from *E. coli* as described before [47]. The recombinant wild type (r-wtGNE) and mutant GNE proteins (r-F307C-GNE (epimerase) and r-A555V-GNE (kinase) [8] were partially purified and epimerase activity was determined in the presence and absence of LTC-181 or LTC-1717 using the Morgan and Elson method [4, 16, 44]. As shown in Fig. 4a, the epimerase activity of r-F307C-GNE and r-A555V-GNE mutant proteins was reduced by ~ 62% compared to r-wtGNE protein. However, treatment of the mutant proteins with 1µM compound LTC-181 and LTC-1717 enhanced epimerase activity of r-F307C-GNE mutant protein by ~ 1.41 fold and ~ 1.91 fold, respectively. Similarly, the epimerase activity of r-A555V-GNE was increased by ~ 1.79 and ~ 2.12 folds after the treatment with LTC-181 and LTC-1717, respectively (Fig. 4a). This study indicates that LTC-1717 and LTC-181 significantly enhanced the activity of recombinant GNE mutant proteins in vitro.

The effect of these compounds was further analyzed by determining the epimerase activity of GNE protein in muscle cell-based model. The cell lysates from L6 WT rat skeletal muscle cell line and SKM-GNEHz (GNE exon 3 heterozygous knockout cell line) were obtained after the treatment of LTC-181 and LTC-1717 as described in Methods. The epimerase activity of endogenous GNE protein from L6 WT and knockout (SKM-GNEHz) cell lines was determined using Morgan and Elson assay. As shown in Fig. 4b, a 44% reduction in epimerase activity was observed in the SKM-GNEHz cell line compared to the L6 WT control owing to the presence of single functional allele. Previous studies suggested the reduction in the transcript levels of GNE in SKM-GNEHz cell Line due to knockout of exon 3 at one allele [14]. Treatment of SKM-GNEHz with LTC-181 increased the GNE epimerase activity by 2-fold, and treatment with LTC-1717 significantly increased the epimerase activity of GNE by ~ 2.51 fold (Fig. 4b). Thus, LTC-1717 significantly increased the epimerase activity of the endogenous GNE protein present in the skeletal muscle cell while a trending increase was observed after LTC-181 treatment. It is of interest to mention that these heterozygous knockout cell lines may not fully capture the extent of dysfunction seen in patient muscle cells. However, lack of primary cells or available animal model limits the data procurement from these KO cells. Therefore, these results will be further validated in animal models.

Since GNE is involved in sialic acid biosynthesis, reduction in epimerase activity affects sialic acid production in the cell. To determine the effect of these compounds on the total sialic acid content, the cells were subjected to LTC-181 and LTC-1717 treatment followed by total sialic acid determination. As shown in Fig. 4c, there was ~ 20% decrease in total sialic acid of GNE knockout cells compared to L6 WT cells. Treatment with LTC-181 and LTC-1717 led to ~ 2 fold and 2.30 fold increase in total sialic acid content of SKM-GNEHz cells compared to untreated cells (Fig. 4c). This study suggests that treatment of skeletal muscle cells with these compounds may enhance the epimerase activity of GNE leading to restoration of total sialic acid content in GNE deficient cells.

#### Effect of small effector molecules on cell viability

**Fig. 5:**
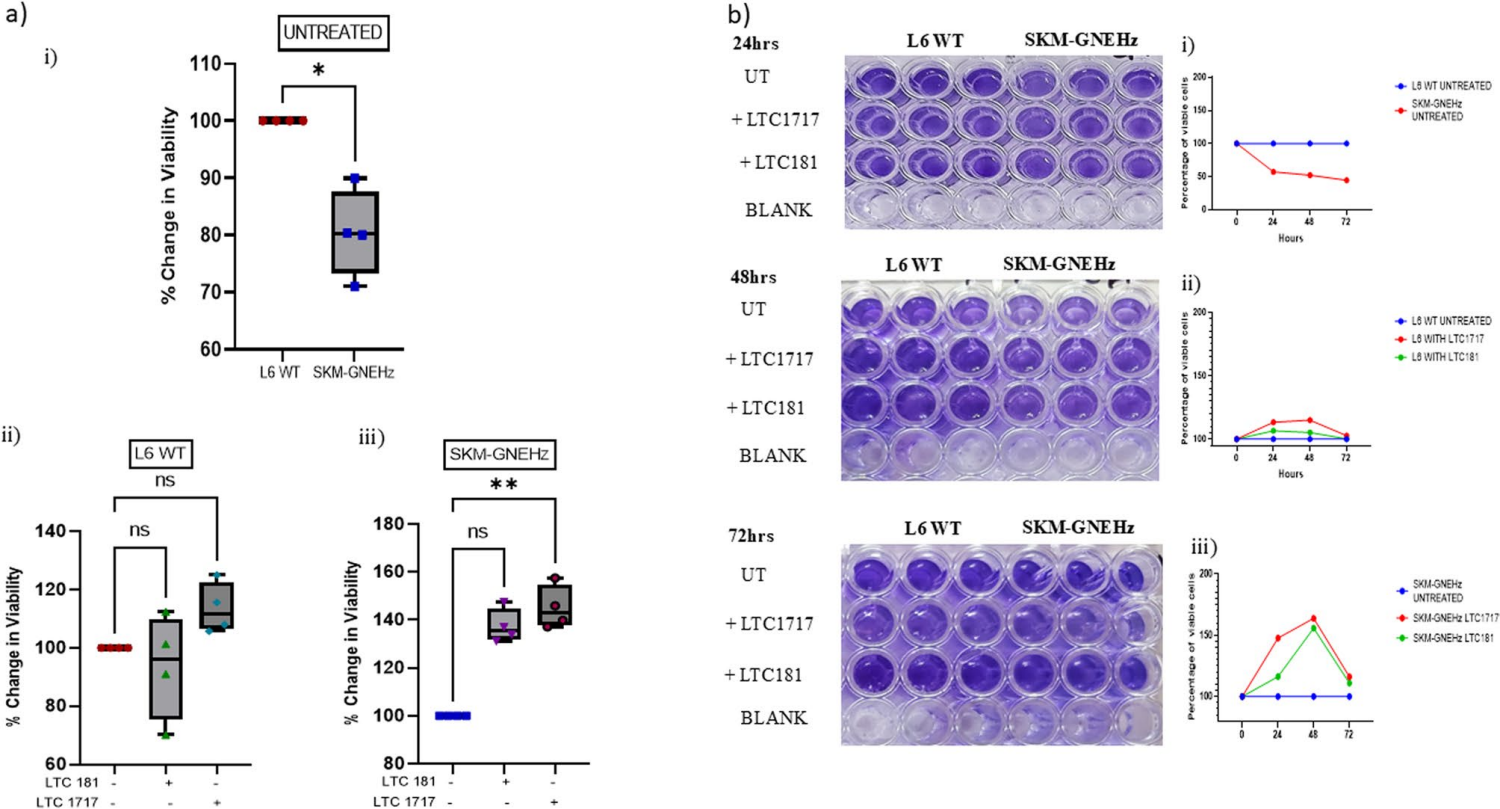
Determination of viable cell count: **a** Graphical representation of viable cells as measured by colorimetric MTT assay. The statistical significance was assessed by Kruskal-Wallis test followed by Dunn’s post-test. **b** Time course of colorimetric assessment of cell survival rate using crystal violet staining as described in methods. i) Untreated L6 WT and SKM–GNEHz cells. ii)L6 WT cells treated with LTC-1717 and LTC-181 iii) SKM–GNEHz treated with LTC-181 and LTC-1717. Two-way ANOVA test for differences between untreated control L6 WT and SKM-GNEHz showed significant differences (***p < 0.001) at 24 h, 48 h incubation. Similarly, SKM–GNEHz cells treated with LTC-1717 and LTC-181 showed significant (****p < 0.0001) compared to untreated at 24 h, 48 h incubation. *n* = 3 biological replicates/triplicates. ‘ns’ represent non-significant and bars represent the mean ± SD; *p < 0.05, **p < 0.01, and ***p < 0.001

The development of small effector molecules as potential drug molecules relies on their effect on the overall viability of the cells. The cells were subjected to MTT assay in which MTT is reduced to purple formazan crystals by NAD(P)H-dependent oxidoreductase enzymes present in viable cells. As shown in Fig. 5a, while treatment of L6 WT cells with these compounds showed no significant change in cell viability, the SKM-GNEHz cell Lines showed 32% and 47% increment in cell viability after treatment with LTC-181 and LTC-1717, respectively, compared to untreated SKM-GNEHz cells. The study suggests that these compounds improved the cell viability of GNE deficient cells (Fig. 5a). The viability of cells was further tested by Crystal Violet Staining (Fig. 5b). A significant reduction (45%^****^, 52%^****^, and 54%^****^ in 24 h, 48 h, and 72 h, respectively) was observed in the viability of SKM-GNEHz cells as compared to L6 WT. Treatment of L6 WT cells with these compounds did not affect the cell viability significantly. However, treatment of SKM-GNEHz cells with LTC-1717 showed significant increase in cell viability (59%^****^, 66%^****^, 8%^ns^ after 24 h, 48 h, and 72 h). Also, treatment with LTC-181 in SKM-GNEHz cells showed trending non-significant increase in cell viability (10%^ns^, 46%^****^, 13%^ns^ after 24 h, 48 h, and 72 h). This study suggests that these molecules were not only non-cytotoxic to cells but were also protective in nature.

#### Effect of small effector molecules on proteomic profile

**Fig. 6:**
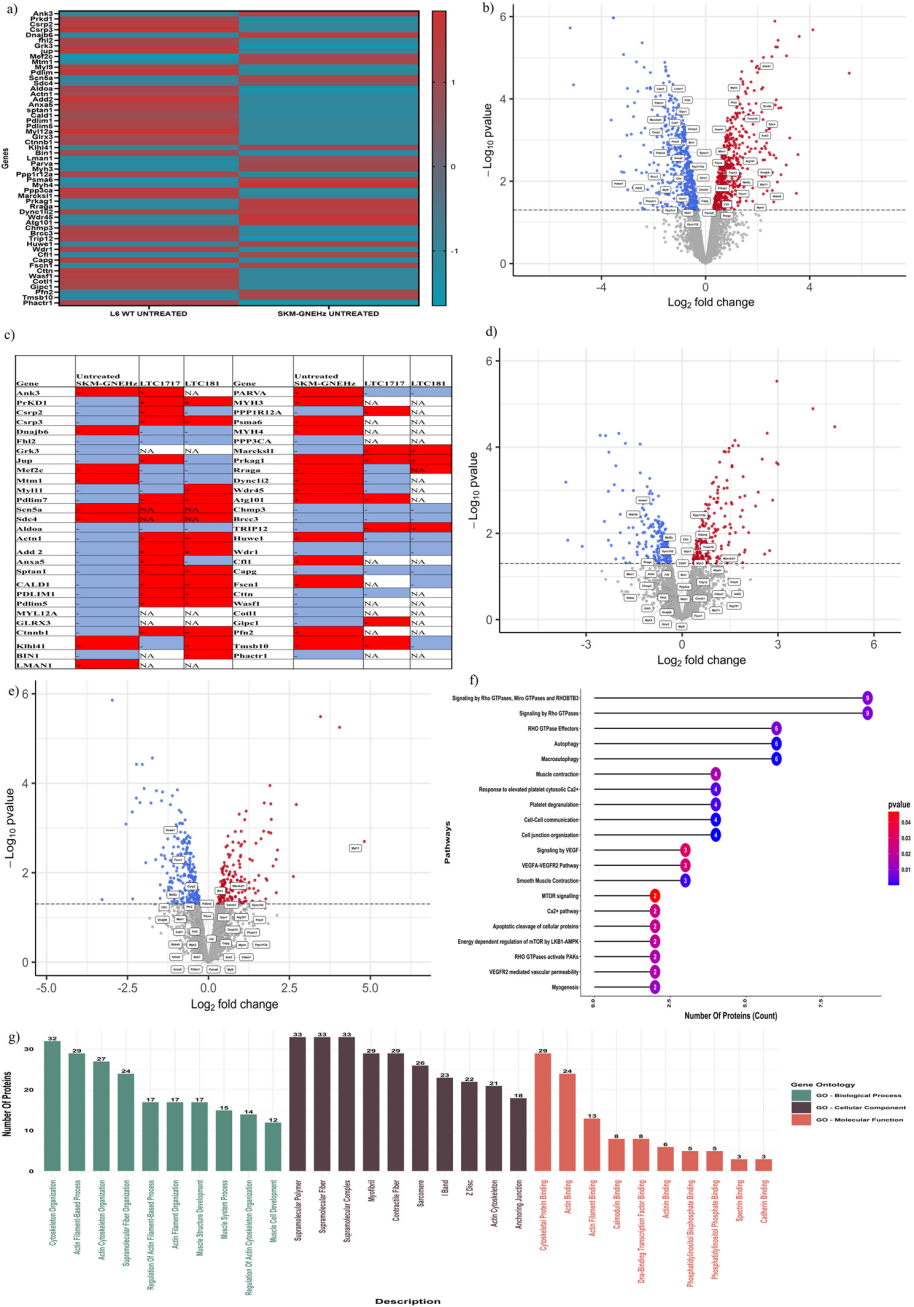
Proteomic profile of L6 WT Vs SKM-GNEHz **a** Heat map of differentially expressed protein in L6 WT vs. SKM-GNEHz cells. **b** Volcano plot of DEGs between L6 WT and SKM-GNEHz. **c** Comparison of differentially expressed genes represented in heatmap of SKM-GNEHz cells before and after treatment with LTC-1717 and LTC-181. (Red – Overexpression, Blue – Down expression) **d** Volcano plot of DEGs between SKM-GNEHz Untreated and SKM-GNEHz LTC-1717. **e** Volcano plot of DEGs between SKM-GNEHz Untreated and SKM-GNEHz LTC-181. The X coordinate was log2 (fold change) and the Y coordinate was − log10 (p value). Each dot represents a gene. Red dots are the upregulated genes of significant expression. Blue dots are the downregulated genes of significant expression. Gray dots are non-significant differentially regulated genes. **f** Pathway enrichment analysis of these genes. **g** The figure presents a representative list of the significantly enriched GO. Bar charts showing the top 10 GO terms for biological process, cellular component and molecular function categories ranked by fold enrichment following analysis

Proteomic studies of L6 WT and SKM-GNEHz cells emphasize changes in proteins of the cytoskeleton and the sarcomere filaments, affecting its maintenance and organization along with certain proteins involved in protein aggregation, autophagy, and muscle atrophy. These findings could point to a role of GNE in the muscle filamentous apparatus, protein aggregation, and muscle fiber autophagy and atrophy that could contribute to GNEM pathogenesis. The altered expression levels of distinct skeletal muscle proteins, as shown in the heatmap in Fig. 6a, might be helpful not only for deepening our understanding of the pathophysiology of GNE myopathy but can also be required for targeted muscle cell-specific therapy. The volcano plots of the differentially expressed genes (DEGs) between L6 WT and SKM-GNEHz is shown in Fig. 6b. Various proteins involved in actin organization and cell migration such as Ank3, Mef2c, Mtm1, Scn5a, Sdc4, Klhl41, Parva, Myh3, Psma6, Myh4, Myl11 were found to be upregulated in SKM-GNEHz compared to L6 WT. Also, various E3 ubiquitin ligases such as Huwe1, Trip12, Autophagy related proteins like Prkag1, Rraga, Wdr45, Atg101, Cfl1, Fscn1, Pfn2, Tmsb10 and stress related proteins such as Dnajb6 were upregulated in SKM-GNEHz as compared to L6 WT. Whereas genes such as Prkd1, Csrp2, Csrp3, Fhl2, Grk3, Jup, Myl9, Pdlim, Aldoa, Actn1, Add2, Anxa5, Sptan1, Cald1, Pdlim1, Pdlim5, Pdlim7, Glrx3, Bin1, Lman1, Ppp1r12a, Dync1li2, Ppp3ca, Marcksl1, Dync1li2, Chmp3, (actin organization and cell migration) Brcc3 (Ubiquitination), Wdr1, Capg, Cttn, Wasf1, Cotl1, Gipc1, Phactr1 (Autophagy related protein) were downregulated in SKM-GNEHz as compared to L6 WT. Figure 6c documented the effect of LTC-1717 and LTC-181 on altered protein expression observed in SKM-GNEHz cells. It was observed that LTC-1717 significanlty modulated the expression of Huwe1, Wdr45, Mef2c, Dync1li2, Rraga, Ppp1r12a, Pdlim5, Tmsb10, Gipc1, Cttn, Marcksl1 and various other non-significant genes. Whereas LTC-181 modulated the expression of Huwe1, Fscn1, Csrp2, Mef2c, Marcksl1, Bin1 significantly and the expression of other genes in a trending manner as indicated in the Volcano Plot for SKM-GNEHz Untreated and SKM-GNEHz Treated (LTC-181 and LTC-1717) as shown in Fig. 6d and e. Gene Ontology (GO) enrichment analysis and Pathway enrichment analysis of these genes were shown in Fig. 6f and Fig 6g. GO analysis showed that DEGs were significantly enriched in biological processes, molecular functions, and cell components, including cytoskeleton organizations, actin filament-based process, muscle cell development, supramolecular fibers, myofibril, cytoskeletal protein binding and actin binding. Some pathways were enriched in the DEGs, such as signaling by Rho GTPases, Autophagy, Cell-Cell communication, Myogenesis etc. The results of the pathway analysis were ranked by enrichment score and the pathways with P-value < 0.05. The study indicates that LTC-1717 and LTC-181 may support the cytoskeletal network, autophagy and atrophy pathway to help overcome the GNE defect.

#### Effect of small effector molecules on alternate roles of GNE

Besides sialic acid biosynthesis, GNE has been shown to affect other cellular functions such as cell migration, ER Stress, autophagy, apoptosis, and protein aggregation. The clinical trials based on sialic acid supplementation could not cure the disease entirely, rather it slows the disease progression only, suggesting role of GNE in other pathways. To investigate if these compounds support GNE in other cellular functions, we determined the effect of LTC-181 or LTC-1717 on cell migration, autophagy and atrophy phenomenon.

#### Effect of small effector molecules on actin dynamics and cell migration

**Fig. 7:**
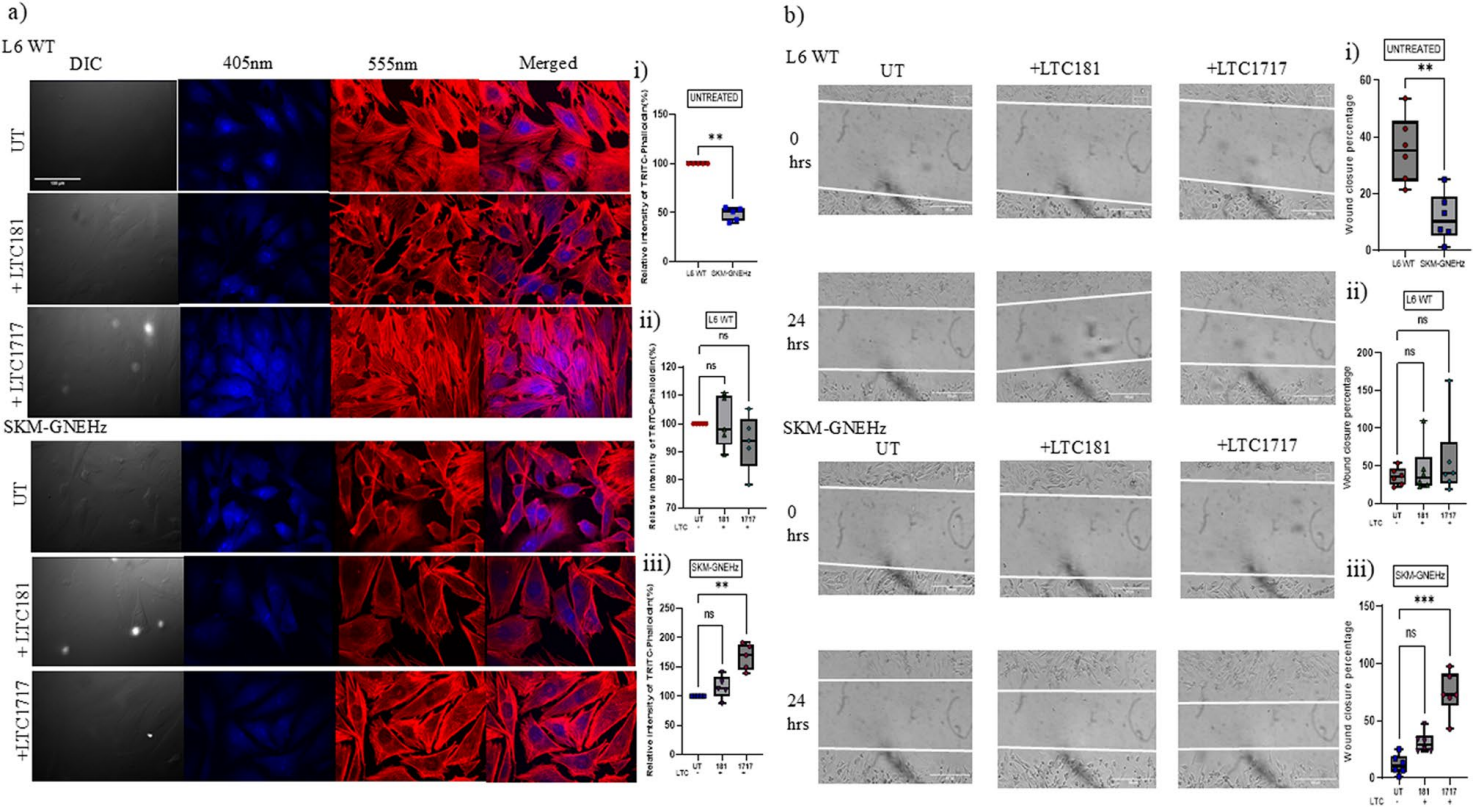
Effect of small molecules on actin and cell migration: **a** *Visualization of F-actin filaments* using TRITC-Phalloidin staining via confocal microscopy in L6 WT and SKM-GNEHz cells before and after treatment with compounds. Scale bar = 100 μm. Quantitative representation of F-actin TRITC Phalloidin staining using Image J software. **b** *Cell Migration Assay*: Wound was created in L6 WT and SKM-GNEHz cells and wound closure percentage was calculated before and after the treatment with compounds as described in methods. Scale bar = 500 μm. (i) Untreated L6 WT and SKM-GNEHz cells. (ii) L6 WT cells treated with LTC-181 and LTC-1717 (iii) SKM-GNEHz treated with LTC-181 and LTC-1717. The statistical significance was assessed by Kruskal-Wallis test followed by Dunn’s post-test and bars represent the mean ± SD; *n* = 6 biological replicates. *p < 0.05, **p < 0.01, ***p < 0.001 and ****p < 0.0001. ‘ns’ represent non-significant

Since role of GNE in cytoskeletal organization was shown to affect F-actin polymerization via Rho and Cofilin signaling proteins in β1 integrin signaling cascade, we investigated the effect of these compounds on F-actin polymerization through Phalloidin staining. As shown in Fig. 7a, the F-actin polymerization was completely disrupted in GNE knockout cells. However, treatment with LTC-1717 improved the F-actin striations by 64% and LTC-181 by 12% in SKM-GNEHz cells compared to untreated SKM-GNEHz cells. Further the cell migration pattern of SKM-GNEHz cells significantly increased after treatment with both the compounds as compared to untreated SKM-GNEHz cells (Fig. 7b). The quantitative estimation revealed 2-fold and 5-fold increase in cell migration of GNE knockout muscle cells compared to L6 WT cells after treatment with LTC-181 and LTC-1717, respectively. This data suggests that these compounds could positively affect the cytoskeletal network regulation by improving F-actin polymerization in GNE deficient cells.

#### Effect of small effector molecules on autophagy and atrophy

**Fig. 8:**
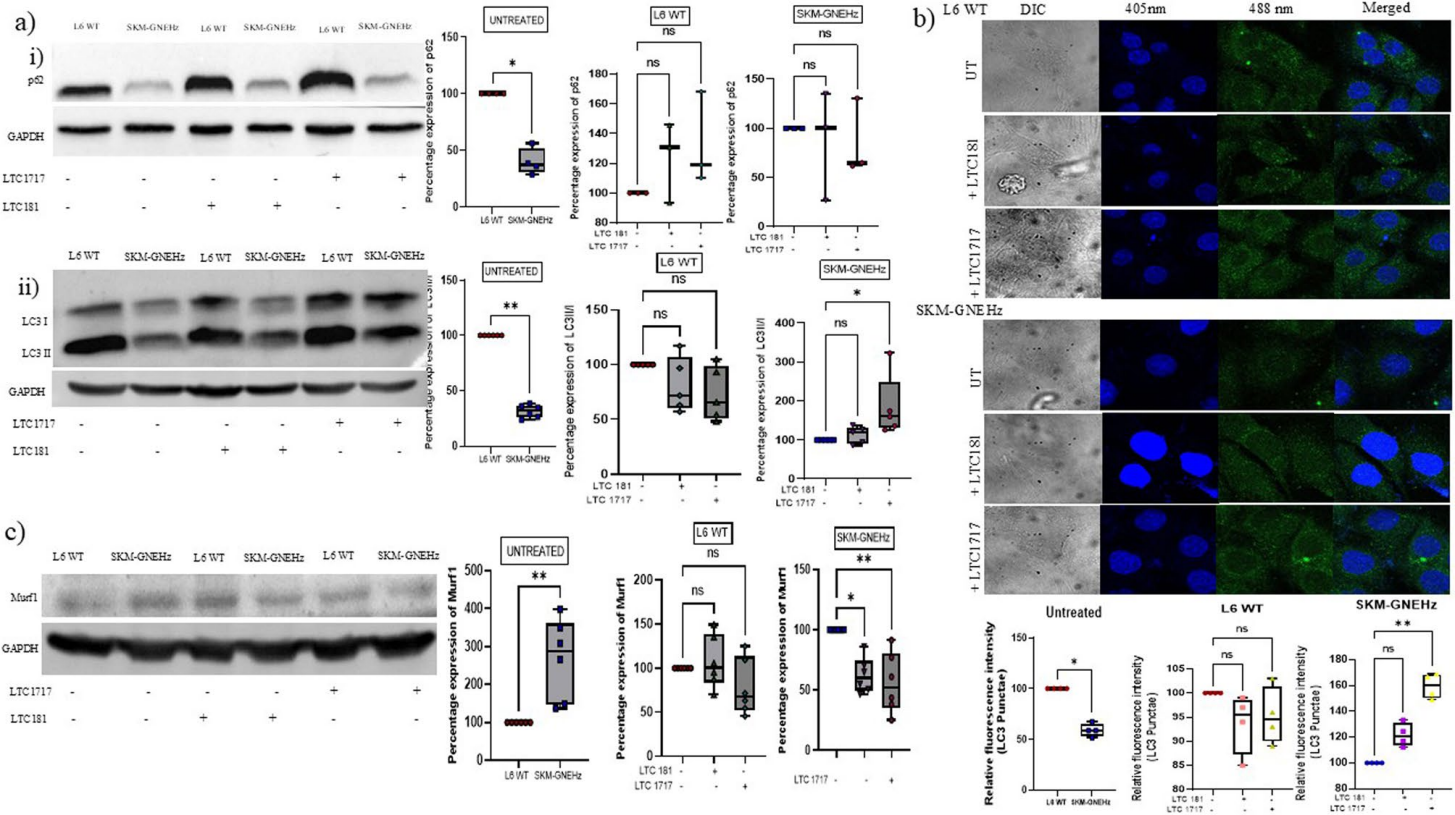
Effect of small molecules on autophagic flux and muscle atrophy: **a** Expression levels of autophagic markers in presence and absence of small molecules-i) p62; ii) LC3I/II; **b** Effect of small molecules on LC3II punctae visualization by confocal microscopy. LC3II punctae visualized under the confocal microscope in basal conditions and quantitative representation of the number of LC3II autophagosomes per cells was plotted. **c** Expression levels of muscle atrophy marker, MuRF1, in presence and absence of small molecules. The statistical significance was assessed by Kruskal-Wallis followed by Dunn’s post-test and bars represent the mean ± SD; *n* = 5 biological replicates. *p < 0.05, **p < 0.01, ***p < 0.001 and ****p < 0.0001. ‘ns’ represent non-significant

The phenomenon of protein aggregation observed in muscle biopsies of GNEM patients could be the consequence of protein misfolding, ER stress, loss of UPR and inability to remove aggregated proteins through autophagy. In order to understand the effect of these compounds on ER stress and protein aggregation, the L6 WT and SKM-GNEHz cells were treated with Mero-GFP for redox potential and Thioflavin S for protein aggregation. No significant effect on ER stress and protein aggregation was observed in GNE deficient cells after treatment with LTC-181 or LTC-1717 (Figures S10-S11; Supporting Information). To determine the effect of these compounds on the phenomenon of autophagy, expression levels of autophagic markers such as p62 and LC3II/I ratio were studied. Significant reduction of approx. 50% and 70% was observed in expression levels of p62, LC3II/I ratio in SKM-GNEHz cells compared to L6 WT cells suggestive of abnormal autophagic flux (Fig. 8a). However, after treatment of SKM-GNEHz cells with LTC-181 and LTC-1717, approx. 13% and 80%* increase in LC3II/I ratio was observed compared to untreated SKM-GNEHz cells, respectively (Fig. 8a). Contrary, no significant increase in p62 protein levels were observed after treatment with these compounds in either L6 WT or SKM-GNEHz cells. Further the effect of these molecules on autophagosome formation was studied by determining LC3II punctae under Confocal microscope. As shown in Fig. 8b significant decrease ~ 40% in LC3II punctae was observed in SKM-GNEHz cells as compared to L6 WT. After the treatment of SKM-GNEHz cells with LTC-181 and LTC-1717, the LC3II punctae increased by ~ 19% and ~ 50% respectively as compared to SKM-GNEHz untreated cells. Our study suggests that these compounds may support GNE function to enhance autophagy by upregulating LC3II/I ratio.

GNE Myopathy is characterized by skeletal muscle weakness and atrophy. The levels of muscle atrophy marker, MuRF1 were analyzed in presence or absence of these compounds. As shown in Fig. 8c, MuRF1 is significantly upregulated in SKM-GNEHz cells as compared to L6 WT untreated cells(~ 3folds). However, treatment with LTC-1717 and LTC-181 resulted in ~ 0.61 folds and ~ 0.55 folds reduction in MuRF1 levels in SKM-GNEHz cell lines compared to untreated cells (Fig. 8b). Our study indicates that these compounds may significantly reduce muscle atrophy observed in GNE deficient cells.

## Discussion

GNE Myopathy belongs to a group of heterogeneous rare orphan muscular disorders whose treatment options have not been approved anywhere in the world. Lack of effective treatment is primarily attributed to the limited number of patients, slow disease progression and quantitative clinical outcomes of the disease. Also, pharmaceutical companies find these treatments less appealing due to their high development costs in comparison to their prospective market size. Currently approved therapies for various rare diseases include gene therapy, oligonucleotide therapy, antibody and protein replacement therapy as well as small molecule drugs [51]. With the advantage of low cost, multiple routes of administration, stability, scale of synthesis and controlled dosing, small molecule drug development is a preferred therapeutic option. For GNE Myopathy, supplementation with sialic acid and its precursors, ManNAc, showed promising results upto Phase 2 clinical trials [50]. However, lack of statistical significance in the cohort of patient study for Phase 3 clinical trials forced the clinicians to reconsider the therapeutic molecule [33]. In the present study, we have identified a potential small effector molecule that helps in rescuing GNE functional defects by enhancing its enzymatic activity and other cellular signaling pathways.

Among the various classes of organic synthesis molecules, HEA and phthalimides emerged as a promising pharmacological lead with low toxic effects and pleiotropic effect on the metabolism of macromolecules [11]. HEA and Pht derivatives have been shown to be effective in the treatment of various neurodegenerative disorders such as Alzheimer’s. Pht compounds have demonstrated anti-tumor activity by inhibiting proliferation and formation of autophagosomes [31]. We have synthesized two HEA derivative compounds, LTC-1717 and LTC-181, which possess bioactive heterocyclic scaffolds.

Closure of translational gaps for therapeutic development requires extensive testing of proposed small effector molecules on cellular models and biological phenotypes. The effect of LTC-1717 and LTC-181 on enzymatic activity of GNE was demonstrated by in vitro and in vivo assays. While both the compounds enhanced the epimerase activity of GNE, LTC-1717 showed significant increase in enzyme activity that led to increased sialic acid content of GNE mutant cells. Loss of epimerase activity of GNE leading to hyposialylation of proteins in GNE deficient cells is considered to be an important pathological defect observed in the GNEM patients. In a previous study by Sharma et al., kinase activity in GNE mutant cells was assessed by monitoring the decrease in NADH absorbance and was shown to remain unchanged, indicating that mutations may not significantly impair kinase function [47]. Reports also suggest that kinase mutations of GNE may be supplemented by alternate kinase enzyme N-acetylglucosamine kinase (NAGK) present in the cell [39]. However, defects in epimerase function of GNE may be lethal. Possibly minimal threshold functional epimerase activity is required for proper functioning of the cell. Wild type cells produce optimal functional GNE protein while heterozygous GNE KO cells lack the minimal requirement of functional GNE. Therefore, treatment of KO cells with a compound enhancing the epimerase activity or stabilizing the functional GNE could rescue the cellular defect due to GNE deficiency. Thus, restoration of sialic acid content post-treatment with LTC-1717 suggest that restoration of sialylation levels may be helpful in combating other cellular defects.

According to recent research, GNE is involved in processes other than sialic acid metabolism such as protein aggregation, apoptosis, ER stress, autophagy, and cytoskeletal organization [38]. The proteomic profile of L6 WT and GNE knockout (SKM-GNEHz) cell lines showed effect on cytoskeletal network, autophagy and atrophy proteins. Treatment with LTC-1717 showed significant restoration of differentially expressed proteins in GNE knockout cells (SKM-GNEHz) compared to L6 WT cells. Both the derivatives were found to support cell viability and were non-toxic to cells. However, there is a need to address toxicology studies and ensuring specificity of these molecules in the future studies, which is currently beyond the scope of this paper. Previous reports suggest hyposialylation of β1 integrin leads to altered actin dynamics and disruption of F-actin in GNE deficient cell models. Treatment with LTC-1717 restored F-actin polymerization and cell migration properties of GNE deficient muscle cells. Actin dynamics is very critical for cell function to regulate cytoskeleton network, cell division, membrane organization and sarcomere function. F-actin and α-actinin-1 binding prevent tropomyosin binding to F-actin, and disassemble F-actin polymers [13]. Interestingly GNE was found to interact with α-actinin-1 (actin crosslinking protein) in vitro and α-actinin-2 (Z-disk stabilizing protein in muscle contraction), and a mutation in GNE strengthen the bond between GNE and α-actinin-2 [2]. Other proteins of cytoskeleton such as Myosin, Tubulin, Myotilin, Vimentin, Zyxin, Nexilin, and Troponin were found to be differentially expressed in GNE myopathy muscle biopsy samples [46]. Thus, restoration of F-actin dynamics and cytoskeletal network proteins after treatment with LTC-1717 could provide a rationale for therapeutic lead molecule consideration.

Pathologically, GNE myopathy is characterized by the presence of autophagic rimmed vacuoles having deposits of β-amyloid, α‐synuclein, tau, and TDP‐43 proteins [43]. The accumulation of misfolded proteins may result from ER stress, failure of UPR response and autophagy phenomenon to remove insoluble protein aggregates. The rimmed vacuoles stained positively for acid phosphatase in GNE knockout mice expressing human *Gne*^(−/−)^h*GNE*D176V-Tg suggesting initiation of autophagic phenomenon in mice [36]. Downregulation of autophagic markers has also been observed in muscle biopsies of GNEM patients [58]. Treatment of GNE deficient muscle cells with LTC-1717 enhanced the expression of LC3 II, autophagic marker suggesting that LTC-1717 may help in upregulating autophagy phenomenon to remove protein aggregates.

One of the main clinical manifestations of GNE Myopathy patients is muscle weakness and muscle atrophy. Muscle atrophy precedes other cellular changes leading to rimmed vacuole formation as shown in *Gne*^(−/−)^h*GNE*D176V-Tg mouse model [35]. Upregulation of muscle atrophy markers MuRF1 and Atrogin-1 has also been reported in GNEM mice model [9]. In the present study, treatment with LTC-1717 significantly reduced the expression of MuRF1 in GNE deficient muscle cells that may contribute towards reducing muscle atrophy. Our study suggests that the proposed HEA compound may be helpful in combating the cellular defects observed in GNE deficient muscle cells and establish cellular homeostasis.

The question remains how LTC-1717 enhances GNE epimerase activity. Bioinformatic analysis involving molecular docking and MD simulation studies revealed that LTC-1717 stabilizes the mutant GNE protein, thereby increasing the functional output of the enzyme. The SPR analysis also showed that LTC-1717 has a high binding affinity for wild type GNE. Based on the effect of these HEA compounds on GNE enzyme activity and cellular functions that enhance cytoskeletal stability and lessen muscle wasting, we propose to explore them as therapeutic lead molecules for the treatment of GNE Myopathy, a rare neuromuscular disorder. Despite LTC-1717`s greater efficacy in some tests, LTC-181 also yielded quantifiable gains in cell survival, sialic acid restoration, and epimerase activity. For lead optimization, the two compounds offer complementary starting points since they represent different pharmacophores. Keeping both scaffolds in place at this point improves the chances of creating small-molecule modulators of GNE activity that work, especially considering complex clinical challenges associated with GNE myopathy.

## Supplementary Information

Supplementary Material 1.

## Data Availability

No datasets were generated or analysed during the current study.
