# Supplementary material for: Hydroxyethylamine & phthalimide analogs restoring defects due to GNE dysfunction: rare disease therapeutic significance
Source: Mol Med. 2025 Dec 3;31:334. doi: 10.1186/s10020-025-01377-1 (PMC12673733; doi:10.1186/s10020-025-01377-1)
Supplement: Supplementary file 1 — Supplementary Material 1. [file 10020_2025_1377_MOESM1_ESM.docx]

**Hydroxyethylamine & Phthalimide** **analogs** **restoring defects due to GNE dysfunction: Rare Disease Therapeutic Significance**

**Shagun Singh^1^, Meenakshi Bansal^2^, Neha Sharma^2^, Vikas Yadav^3^, Fluencephila Mashangva**^1^**, Jyoti Oswalia^1^, Vaishali Gautam^1^, Gagan Deep Jhingan^4^, Naidu Subbarao^3^, Brijesh Rathi^2*^ and Ranjana Arya^1**^**

^1^School of Biotechnology, Jawaharlal Nehru University, New Delhi-110067

^2^HG Khorana Centre for Chemical Biology, Department of Chemistry, Hansraj College, University of Delhi, Delhi-110007

^3^School of Computational and Integrative Sciences, Jawaharlal Nehru University, New Delhi – 110067

^4^ VProteomics, Green Park, New Delhi - 110016

* Co-Corresponding Author

**Corresponding Author

Mailing Address: School of Biotechnology, Jawaharlal Nehru University, New Delhi- 110067, India

Tel.: +91-11-26738754

FAX: +91-11-26742040

Email: [ranjanaa@mail.jnu.ac.in](about:blank); [arya.ranjana24@gmail.com](about:blank) (ORCID ID: 0000-0001-5924-9351); [brijeshrathi@hrc.du.ac.in](mailto:brijeshrathi@hrc.du.ac.in); [nsrao@mail.jnu.ac.in](mailto:nsrao@mail.jnu.ac.in) (ORCID ID: 0000-0002-1720-2165); [shagunsingh0498@gmail.com](mailto:shagunsingh0498@gmail.com) (ORCID ID: 0009-0005-0843-385X); [nehasharma0024@gmail.com](mailto:nehasharma0024@gmail.com); [meenakshibansal0502@gmail.com](mailto:meenakshibansal0502@gmail.com); [vikas98_sit@jnu.ac.in](mailto:vikas98_sit@jnu.ac.in) (ORCID ID: 0009-0000-9158-6702); [fluence111@gmail.com](mailto:fluence111@gmail.com) (ORCID ID: 0000-0002-3154-3484); [oswalia.jyoti@gmail.com](about:blank) (ORCID ID: 0000-0001-7760-760X); [vaishaliisback@gmail.com](mailto:vaishaliisback@gmail.com); [info@vproteomics.com](mailto:info@vproteomics.com)

**Corresponding Author:** Dr. Ranjana Arya, 127, School of Biotechnology, Jawaharlal Nehru University, New Delhi- 110067, India. Tel.: +91-11-26738754

**Additional Information: -**

**Methods: -**

**Sources of materials and chemicals**

Solvents and reagents were utilized for the experiments without additional purification. Thin-layer chromatography (TLC) on alumina-coated Merck plates was performed with compounds dissolved in chloroform (CHCl_3_) and developed in Ethyl acetate/Hexane (1:1, v/v). The compounds were purified by Flash column chromatography (Yamazen, Japan) using alumina gel columns (100–200 mesh size, CDH) for minor impurities found by iodine vapor/UV light visualization. Microwave reactions were carried out in a sealed vial using the “Start Synth Microwave Synthesis Labstation (Milestone microwave laboratory systems)” microwave apparatus using a power supply of 300 W at 80° C by controlled temperature programming with a 2-minute ramp and holding for 20 min. BUCHI Labortechnik AG CH-9230 was used to measure melting points. Under ambient temperature, an Anton Paar Polarimeter was used to record specific rotation using a 1 percent chemical concentration (1g/100 mL) in ethanol at 589 nm wavelength. Nuclear Magnetic Resonance (NMR) spectra (^1^H & ^13^C) were recorded in CDCl_3_, DMSO, and D_2_O medium on a JEOL ECX-400P NMR at 400 MHz and 100 MHz, respectively, at USIC, University of Delhi. The NMR spectroscopy study utilized Trimethylsilane (TMS) as an internal standard. Chemical shifts (-scale) and coupling constants were measured in parts per million (ppm) and hertz (Hz). Singlet (s), doublet (d), doublet of doublet, triplet (t), quartet (q), and multiplet (m) were the letters written to the splitting patterns (m). A Biosystems Q-Star mass spectrometer also confirmed the product's chemical structures. A High-Performance Liquid Chromatography (HPLC) system equipped with an analytical column (C18) and a Thermo Separation Spectra SERIES UV100 detector paired with software was used to test the purity of the synthesized compounds. The mobile phase was made up of comprised acetonitrile and water (v/v), and the compounds were >95% pure.

**ER-Redox state**

The Mammalian endoplasmic reticulum‐localized redox-sensitive green fluorescent protein (MERO‐GFP) represents a modified form of GFP, engineered to include a mouse Bip signal at the *N*-terminal and an ER localization signal KDEL at the C-terminal of the redox‐sensitive GFP. This variant of GFP undergoes full oxidation upon excitation at a wavelength of 405 nm and complete reduction at 476 nm, resulting in corresponding emissions at 508 nm and 510 nm, respectively.

Transfection of this plasmid into L6 myoblast cells, previously cultured on coverslips within 6-well dishes, was facilitated using Lipofectamine 3000 reagent. Following transfection, cells were subjected to treatment with 0.2 mM DTT for 10 minutes, followed by two washes with 1X PBS and fixation with a 2% formalin solution for 10 minutes. Subsequently, the cells were mounted using 80% glycerol, and images were captured using an Olympus FluoView FV1000 microscope at excitation wavelengths of 405 nm and 488 nm, with a magnification of 60X.

To evaluate MERO‐GFP dynamics, the MERO‐GFP ratio was calculated based on the mean fluorescence intensity at 488 nm and 405 nm excitation wavelengths. This methodology provided valuable insights into the redox status and localization dynamics of MERO‐GFP within the cellular environment.

**Measurement of protein aggregation by Thioflavin S**

For qualitative estimation, 0.5 × 10^5^ cells were grown on a coverslip in 6 well dishes. Upon confluency, cells were washed thrice with 1X PBS and fixed with 3.7% PFA for 20 min at room temperature, followed by washing with 1XPBS three times. Cells were briefly permeabilized (5–30 s) with 0.05% TritonX-100 prepared in PBS and washed thrice with 1X PBS after permeabilization. Blocking with 5% BSA for 20 min was done, followed by 1X PBS wash (3- times). 5 μM Thioflavin S prepared in 1XPBS was added to the cells and incubated for 10 min followed by mounting using DABCO. Slides were observed at 60X magnification with oil immersion objective under laser confocal microscope Olympus FluoView™. For quantitative analysis of aggregation measurement by Thioflavin S, cells were grown in 96-well plate in serum free media for 24 hours followed by washing with 1X PBS and stained with 125 μM of Thioflavin S in PBS for 1 h. The dye was then removed and cells were washed with 1X PBS (3- times). Then fluorescence was recoded with excitation maxima 391 nm, and emission spectra 420-500 nm with slit width of 10 nm.

**Structural Stability and Conformational Changes**

The structures of  Homo sapiens glucosamine (UDP-N-acetyl)-2-epimerase/N - acetylmannosamine kinase (GNE) has been downloaded from RCSB (PDB IDs 4ZHT and 3EO3). To Check the mutational effect on the secondary structures, we first mutate the amino acid PHE at position 307 with CYS in epimerase domain and ALA at 555 with VAL  using the UCSF Chimera software and performed a small energy minimization process using schrodinger followed by md simulation for 300 ns using Gromacs and extracted the lowest energy conformations of proteins.

**Results: -**

**Compound Synthesis and GNE Enzyme activity**

The spectroscopic and spectrometry techniques used to confirm chemical composition of synthesized compounds were shown Figure S1-S7. GNE epimerase enzyme activity in two different GNE mutants F307C and A555V with various compounds is shown in Figure S8 . All compounds except LTC1717 and LTC181 did not show any significant increase in the enzyme activity. A concentration dependent study was performed with LTC1717 and LTC181 with 0.25µM, 0.5 µM, 1 µM, 2 µM, 5 µM, 10 µM,20 µM concentrations of both the molecules. It was observed that maximum epimerase activity is observed at 1 µM as shown in Figure S9.

**Effect of compound LTC181 and LTC1717 on ER Stress in L6 wild type and Knockout cell lines SKM-GNEHz**

ER is maintained as an oxidizing environment for the proper folding of proteins and for regulating ER homeostasis. We determined ER redox in SKM-GNEHz cells by using MERO-GFP (Mammalian Endoplasmic Reticulum-localized Redox-sensitive GFP). The MERO-GFP ratio which is a ratio of fluorescence from excitation 488 nm (reduced state) versus 405 nm (oxidized) was measured in L6 WT cells and SKM-GNEHz cells. As shown in Figure S10, MERO-GFP ratio was found to be significantly higher in untreated SKM-GNEHz cells when compared to untreated L6 WT cells by almost 50% indicating a more reduced ER redox state in SKM-GNEHz cells. We, further, evaluated the effect of LTC181 and LTC1717 on ER redox state in SKM-GNEHz cells. Our result showed a trending decrease in MERO-GFP ratio of treated SKM-GNEHz compared to untreated cells. However, these compounds did not show a significant effect on ER stress suggestive of no direct correlation of these compounds on ER regulated functions.

**Effect of compound LTC1717 and LTC181 on protein aggregation in L6 wild type and Knockout cell lines SKM-GNEHz**

GNE myopathy is characterized by presence of rimmed vacuoles which is aggregation of protein such as β-amyloid, p-Tau, presenilin, α-synuclein, and TDP-43. The protein aggregation in GNE mutant cells was measured by Thioflavin S, a fluorescence benzothiazole salt dye suitable for staining live cells as it easily penetrates into the cell membranes and binds beta-sheet rich moieties of the protein aggregates. This induces a shift in excitation (385–450 nm) and emission (445–482 nm) spectra measured as extent of protein aggregation. Qualitatively, the protein aggregation was measured using Confocal Microscopy which suggests that protein aggregates were concentrated towards perinuclear space in SKM-GNEHz as compared to L6 WT untreated cells. After the treatment with compounds the aggregates were found to be dispersed along the cytoplasm (Figure S 11a). Quantitative detection of protein aggregation with Thioflavin S revealed an increase of 40% in protein aggregation in SKM-GNEHz cells. After treatment with compounds, 8-10% reduction in protein aggregates was observed in SKM-GNEHz cells (Figure S 11b).

**Effect of mutation on GNE Structure**

Fig. S12 shows the RMSD values for wtGNE along with Kinase mutant (A555V-GNE) and Epimerase mutant (F307C-GNE). For the native form, the graph shows initial fluctuations in the backbone, but after approximately 80 ns, the system begins to converge and stabilizes. The overall deviation of atoms remains within an acceptable range, with an average RMSD value of 1.5-2.0 Å, indicating that the protein maintains its structural integrity. In kinase mutant there is no substantial conformational change in the protein due to the single point mutation. However, the epimerase mutant represented by the magenta line, shows a significant increase in RMSD starting at 180 ns, with the value drastically rising to around 7.0 Å by the end of the simulation. This suggests that the single point mutation induced a large conformational change, leading to the destabilization or unfolding of the protein. So, for further Bioinformatics analysis Epimerase mutant r-F307C-GNE was selected.

**Figures: -**

**Figure S1.** ^1^H-NMR spectrum of LTC-181 in CDCl_3_.

**Figure S2.** ^13^C-NMR spectrum of LTC-181 in CDCl_3_.

**
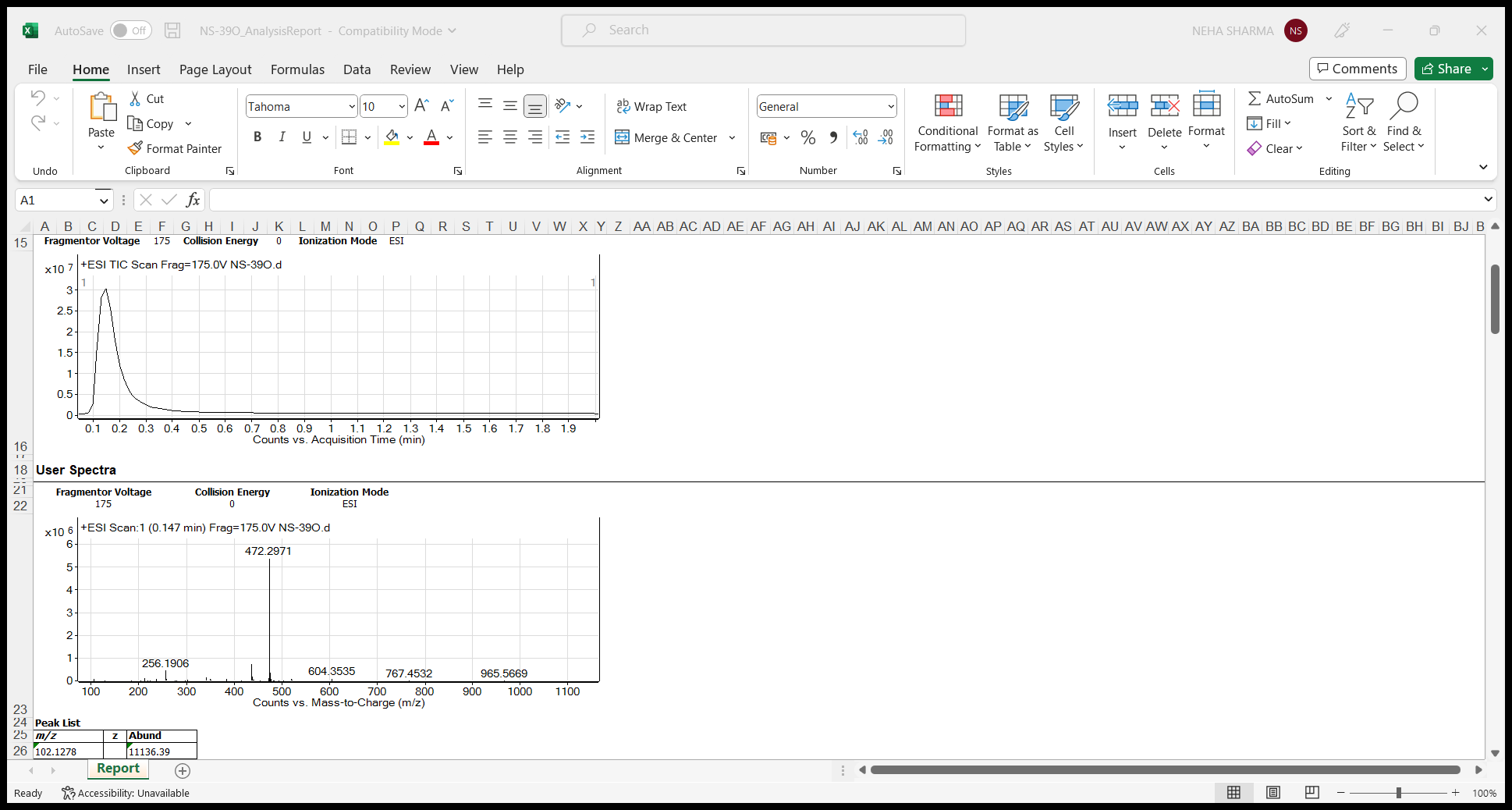
**

**Figure S3.** HRMS spectrum of LTC-181.

**Figure S4.** ^1^H NMR spectrum of compound LTC-1717 in CDCl_3_.

**Figure S5.** ^13^C NMR spectrum of compound LTC-1717 in CDCl_3_.


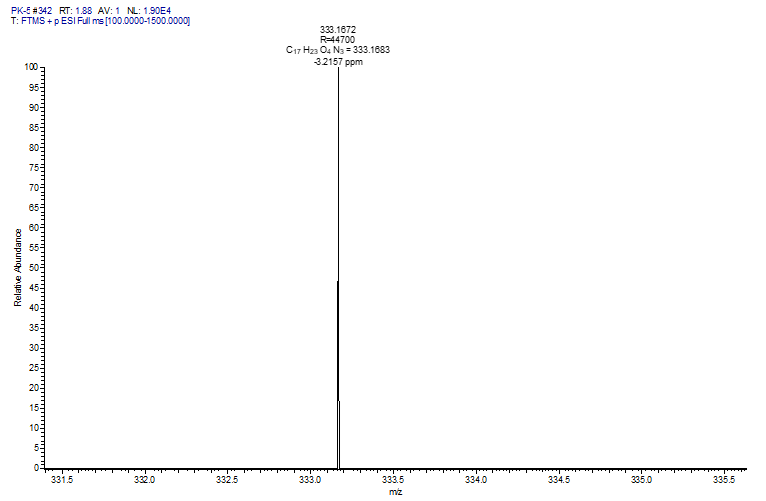


**Figure S6.** ESI (HR-MS) spectrum of LTC-1717.


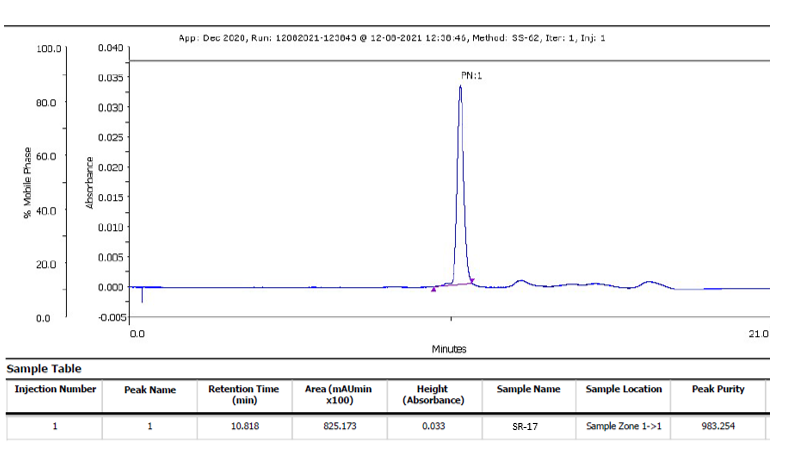


**Figure S7.** HPLC data of compound LTC-1717.


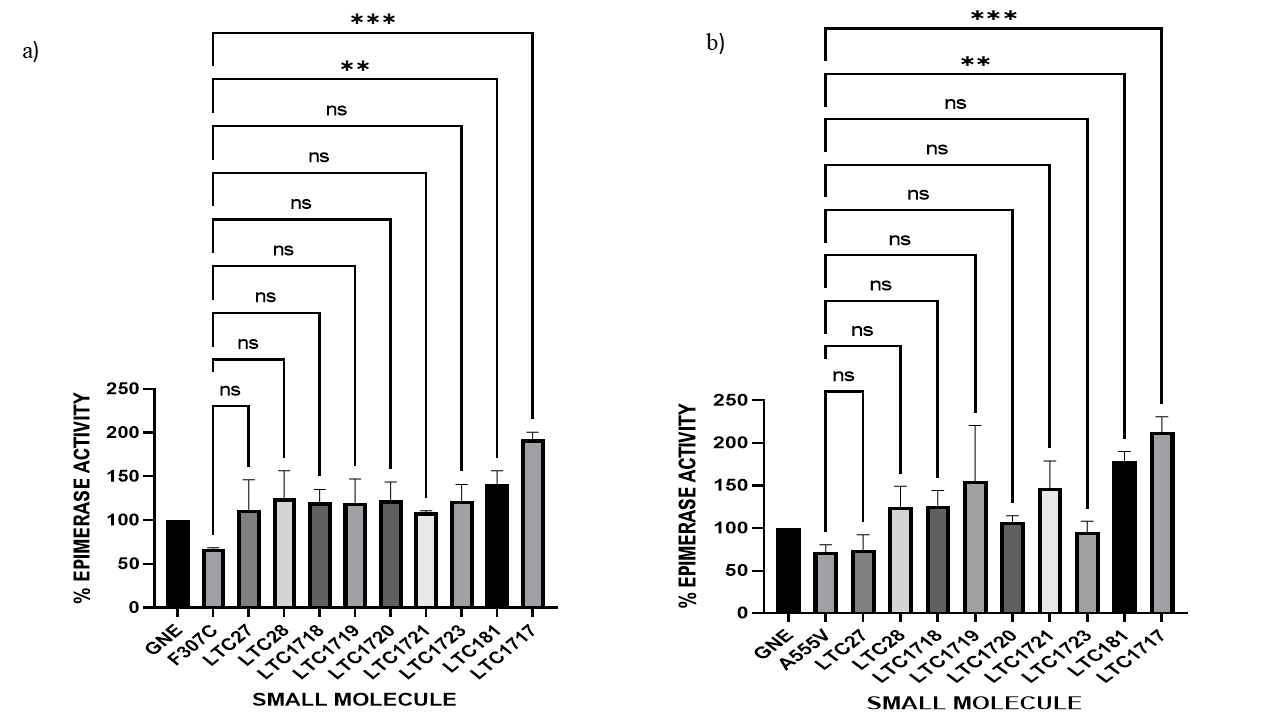


**Figure S8**. *In vitro* Epimerase Activity - Recombinant GNE wild type and mutant protein purified from *E. coli* was used to determine the epimerase activity in presence of small molecules (LTC27, LTC28, LTC1718, LTC1719, LTC1720, LTC1721, LTC1723, LTC181 & LTC1717) using Morgan Elson method. N-acetyl-mannosamine was used as a standard a) Comparison of GNE activity in absence and presence of small moleules with GNE-F307C Mutant b) Comparison of of GNE activity in absence and presence of small moleules with GNE-A555V Mutant. Statistical significance was determined by Kruskal-Wallis test followed by Dunn's multiple comparison post-testand bars represent the mean ± SD; n = 4. ****p < 0.001. ‘ns’ represent non-significant.


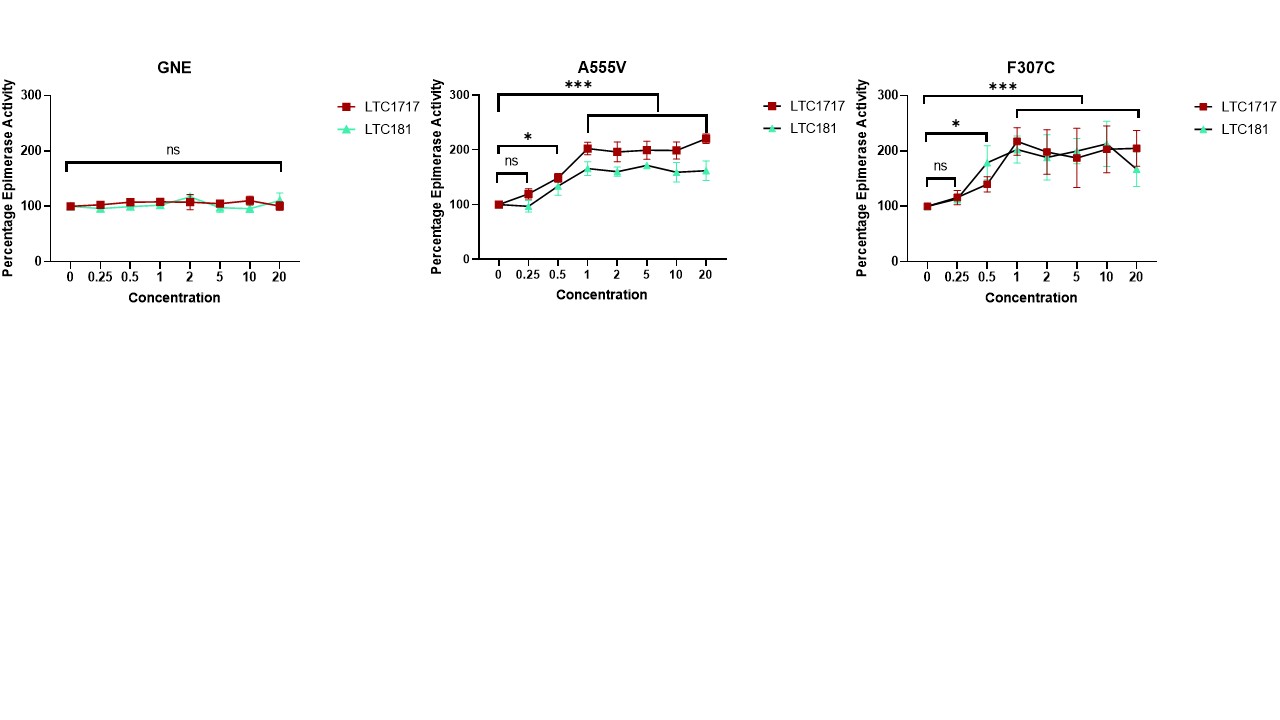


Figure S9. *In vitro* Epimerase Activity - Recombinant GNE wild type and mutant protein purified from *E. coli* was used to determine the epimerase activity in presence of small molecules (LTC181 & LTC1717) at different concentrations (0.25µM, 0.5 µM, 1 µM, 2 µM, 5 µM, 10 µM,20 µM) using Morgan Elson method. Statistical significance was determined by Kruskal-Wallis test followed by Dunn's multiple comparison post-test and bars represent the mean ± SD; n = 4. ****p < 0.001. ‘ns’ represent non-significant.


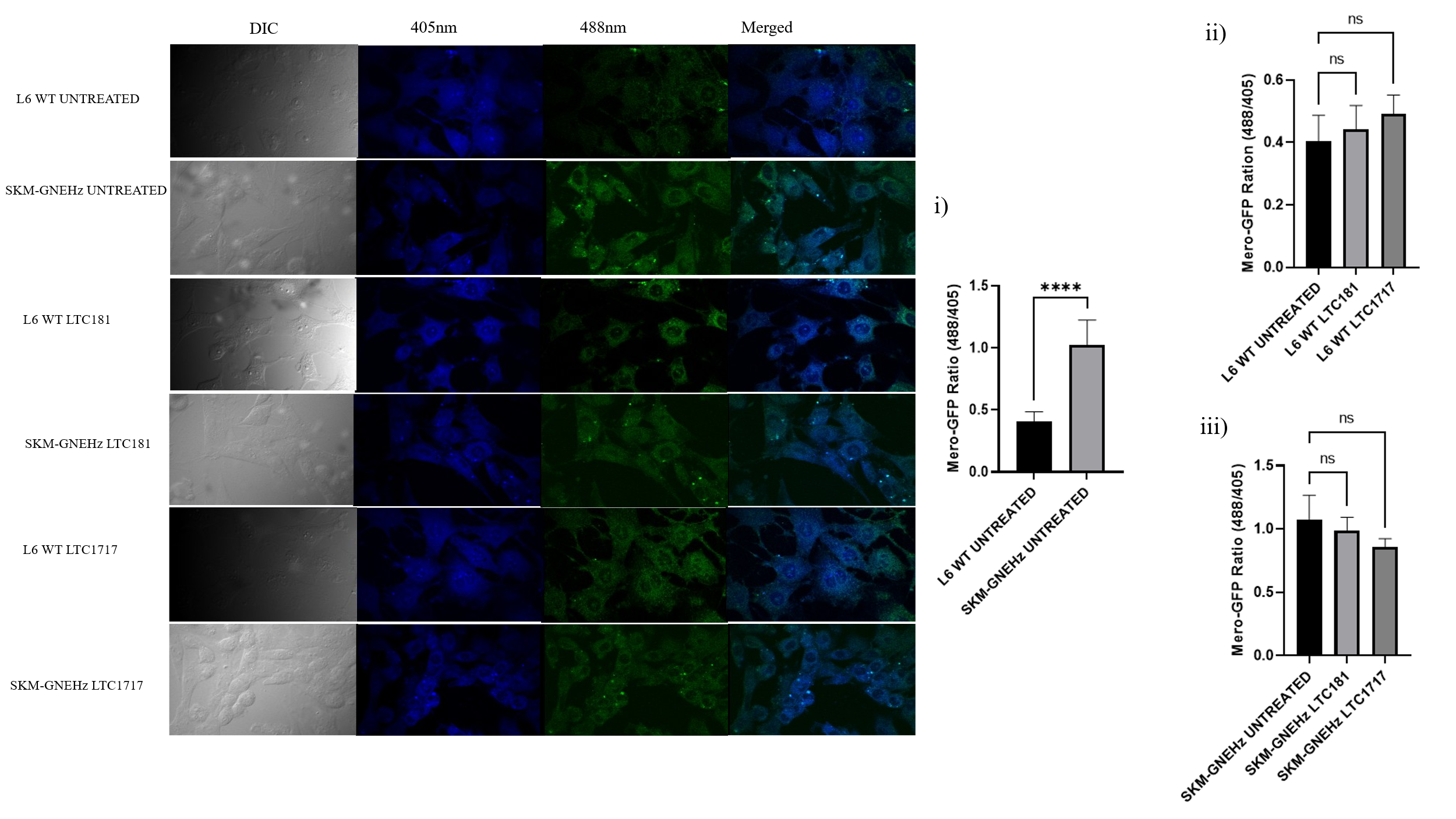
**Figure S10.** Measurement of ER redox state using MERO‐GFP in the absence and presence of LTC181 and LTC1717. Confocal image showing MERO‐GFP expression displaying distinct excitation spectra in the fully oxidized and reduced state, with maxima at 405 and 488 nm, respectively. ER redox state is measured as MERO‐GFP ratio; 488 nm/405 nm. ER stress values in i) Untreated L6 WT and SKM–GNEHz cells. ii) L6 WT cells treated with LTC1717 and LTC181 iii) SKM–GNEHz treated with LTC1717 and LTC181. Statistical significance was determined by Kruskal-Wallis test followed by Dunn's multiple comparison post-test and bars represent the mean ± SD; n = 3. ****p < 0.001. ‘ns’ represent non-significant.


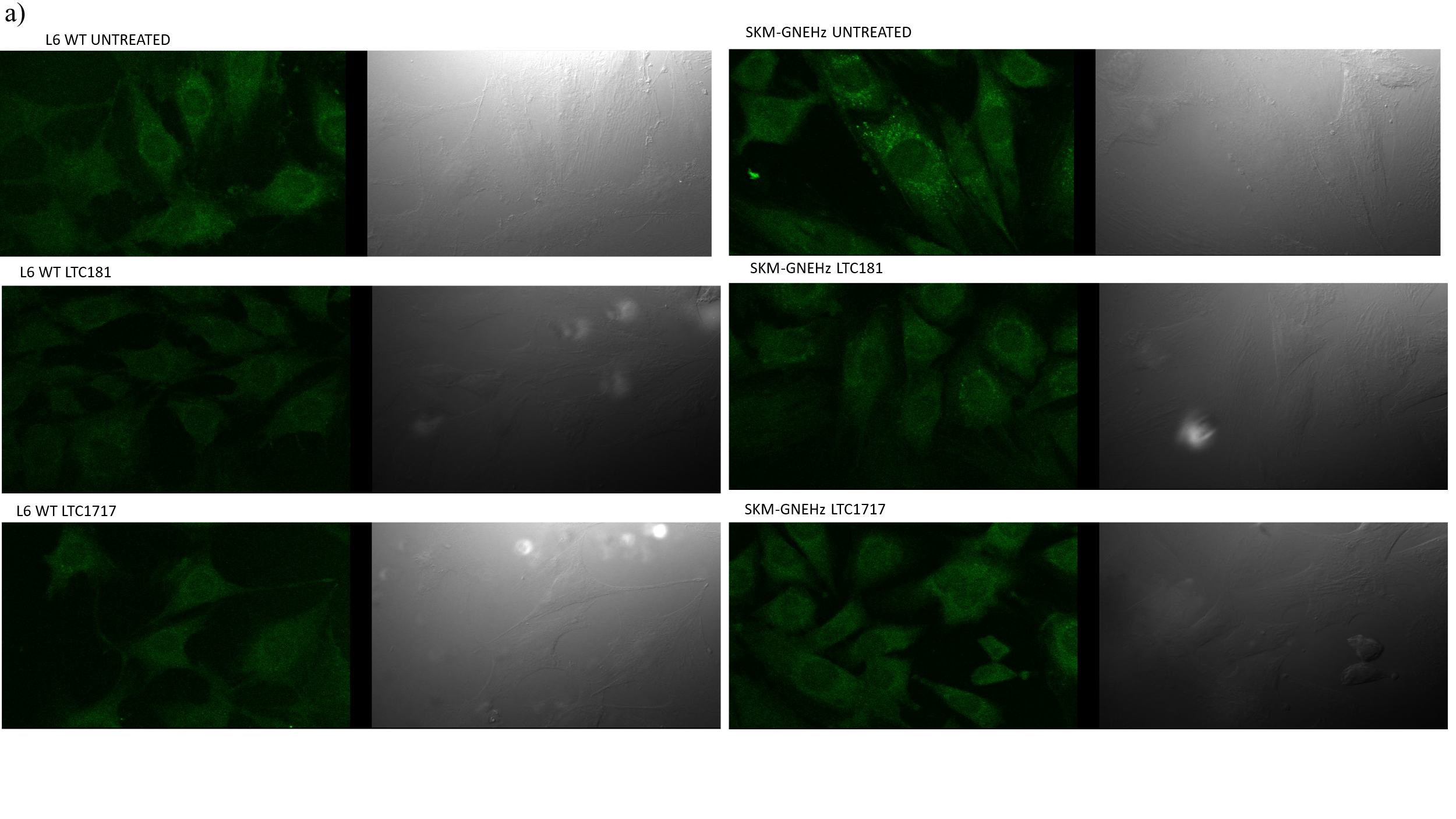


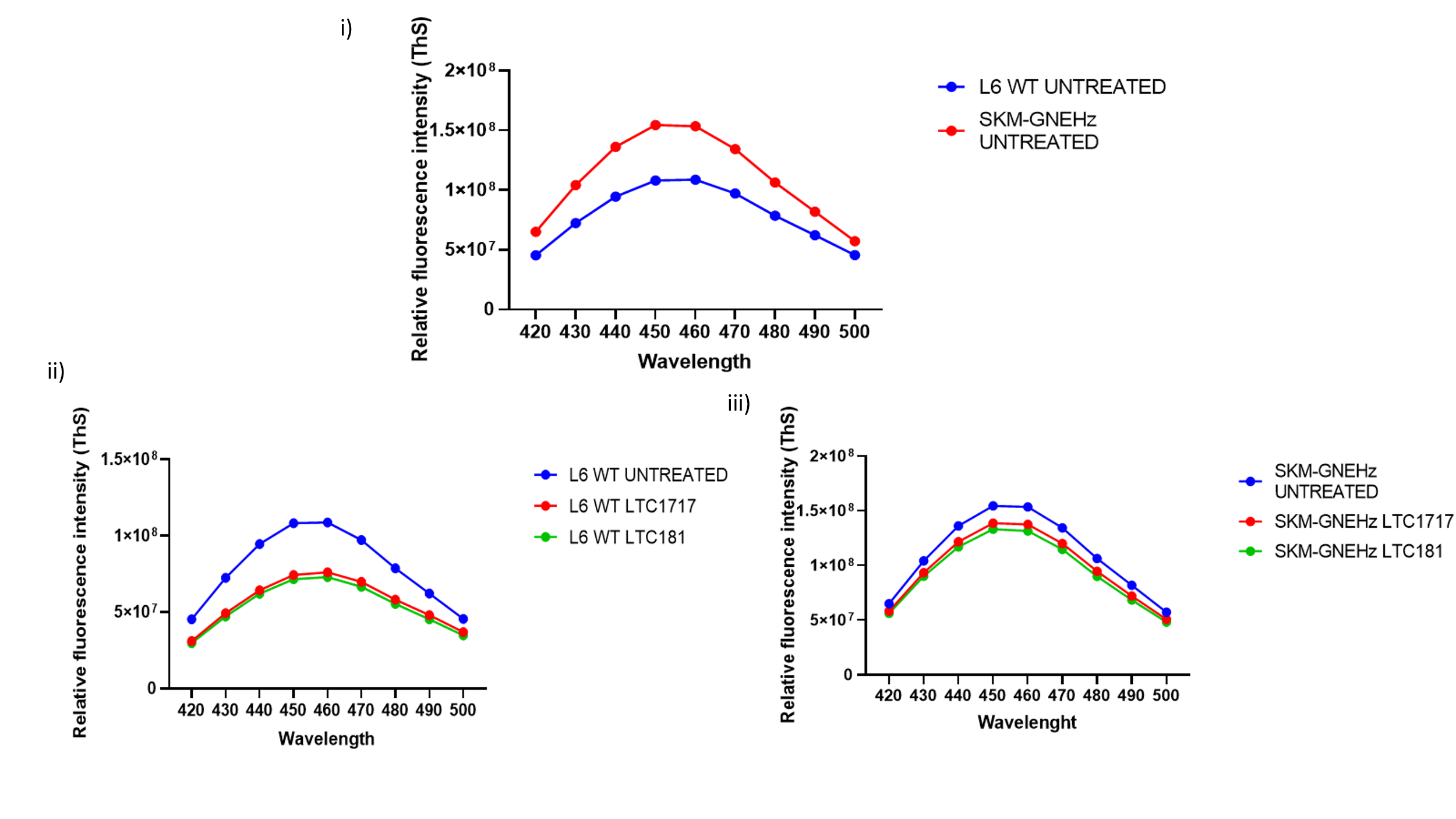
**Figure S11**. a) Effect of LTC1717 and LTC181 on protein aggregation in L6 WT and SKM-GNEHz Cells. Cells were grown in Serum free media for 24 h and fixed with 3.7% paraformaldehyde. The fixed cells were stained with ThS for 10 min and mounted on slides. b) Protein aggregation by ThS using Fluorimeter before and after treatment with LTC1717 and LTC181. Protein aggregation values in i) Untreated L6 WT and SKM–GNEHz cells. ii) L6 WT cells treated with LTC1717 and LTC181 iii) SKM–GNEHz treated with LTC1717 and LTC181. Two-way ANOVA test for differences between untreated control L6 WT and SKM-GNEHz showed significant increase (***p < 0.01). n = 3 biological replicates.


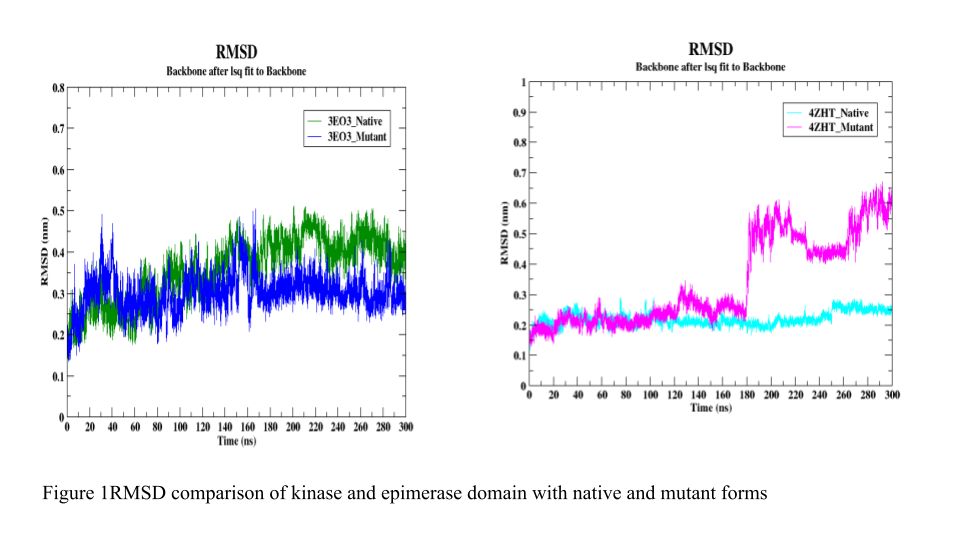
**Figure S12.** RMSD values for wtGNE along with Kinase(3EO3) mutant (A555V-GNE) and Epimerase(4ZHT) mutant (F307C-GNE).


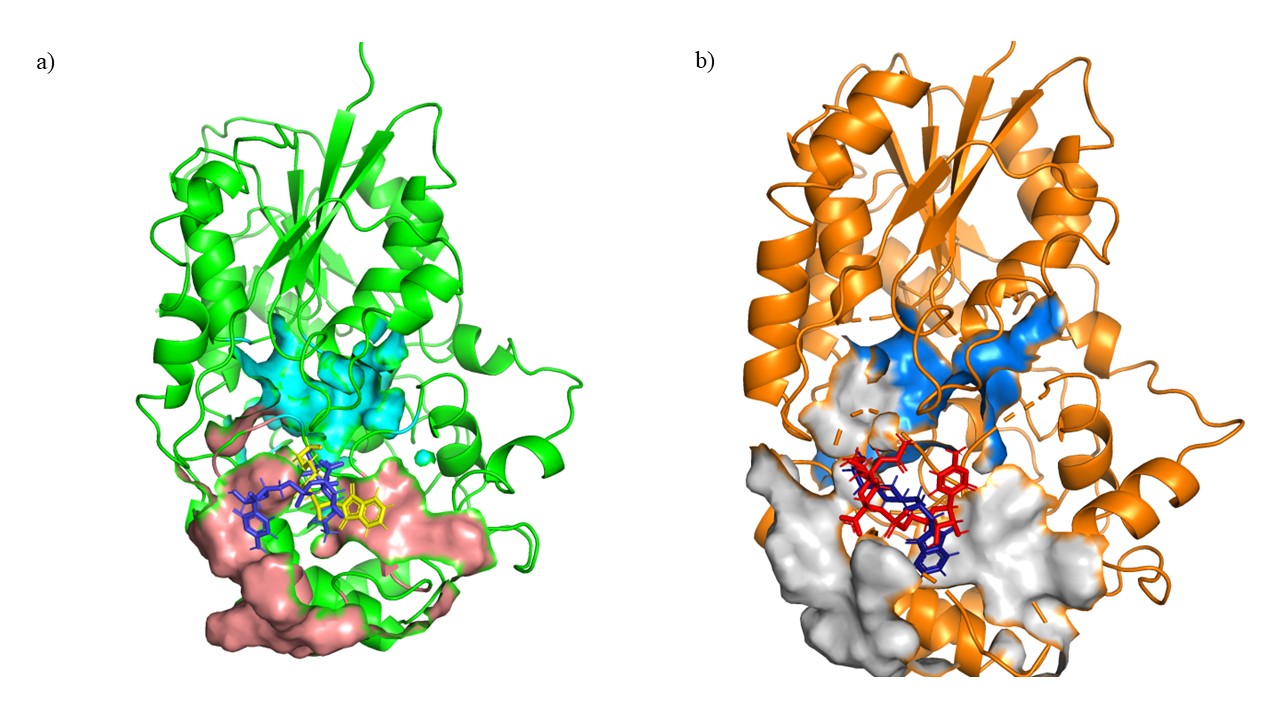


**Figure S13** a) Docking pose of CMP-Sialic Acid (Known compound) represented in blue color, while LTC1717 represented in yellow color at Allosteric site in Salmon color, and UDP-GlcNAc site in Cyan color against 4zht_native. b) Docking pose CMP-Sialic Acid (Known compound) represented in red color, while LTC1717 represented in Dark Blue color at Allosteric site in grey color, and UDP-GlcNAc site in Light Blue color against 4zht_mutant.
